# Developmental analysis of visually evoked defensive behavior identifies age and sex-specific responses and underlying synaptic and glia changes

**DOI:** 10.1016/j.isci.2025.113997

**Published:** 2025-11-11

**Authors:** Georgia Lee Albrecht, Rebekah Ramirez, Delaram Moradpour, Jordan Mar, Rafael Colla Fortes, Matthew A. McGregor, Vishnuvasan Raghuraman, Isabella Farhy-Tselnicker

**Affiliations:** 1 Department of Biology, Texas A&M University, College Station, TX 77843, USA; 2 Texas A&M Institute for Neuroscience (TAMIN), Texas A&M University, College Station, TX 77843, USA; 3 Center for Biological Clocks Research, Texas A&M University, College Station, TX 77843, USA; 4 Department of Computer Science and Engineering, Texas A&M University, College Station, TX 77843, USA

**Keywords:** Neuroscience, Behavioral neuroscience, Molecular neuroscience, Cellular neuroscience, Sensory neuroscience

## Abstract

Defensive behaviors such as freezing and escaping are crucial for survival, involving the complex integration of sensory and motor circuits. While instinctive, the ability to discern threat and adapt responsiveness is acquired through life experience, shaped by sensory maturation and emotional state. Disrupting these processes by sensory deprivation or chronic stress can induce disproportionate reactions, underscoring the need for a comprehensive understanding of age and sex-specific variations in defensive responses and adaptation. Here, we employed behavioral testing and immunohistochemistry to analyze visually evoked defensive responses and synaptic and glial development in the superficial superior colliculus (sSC) and dorsal periaqueductal gray (dPAG). Our findings demonstrate distinct age and sex-dependent behavioral profiles that are correlated with synaptic and glial changes in these brain regions. This study provides critical insights into the establishment and adaptation of visual threat responses and underlying circuits, laying the groundwork for future investigations into the cellular mechanisms governing defensive behaviors.

## Highlights

• Mice responses to a novel visual threat increase with age, peaking in adulthood
• Behavioral adaptation to the visual looming threat is rapid and long-lasting
• Defensive response types such as freezing and escaping are age and sex dependent
• Changes in synapses, astrocytes, and microglia in sSC, dPAG correlate with behavior

## Graphical abstract

**Figure:**
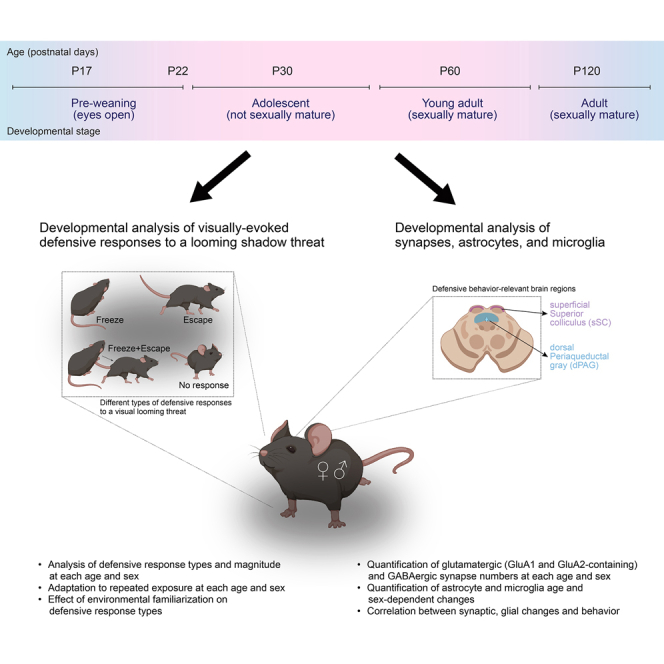

## Introduction

Defensive responses to a visual threat are innate behaviors conserved across multiple species, including rodents and humans of both sexes, and involve the integration of sensory and motor circuits to elicit behaviors such as freezing and/or escaping.^1^^,^^2^^,^^3^^,^^4^^,^^5^^,^^6^ While innate behaviors are instinctive responses that do not require prior experience, discerning between threatening and harmless environmental stimuli must be acquired during the animal’s development and through life experience and is critical for maintaining normal function (such as food foraging) necessary for survival.^7^^,^^8^ Developing animals must memorize the spatial location of a shelter to facilitate effective escape from danger,^9^^,^^10^ while also adapting to repeated environmental stimuli by modifying their responsiveness.^11^ Furthermore, visually triggered responses are tightly coupled with the maturation of the visual circuits, which in mice occurs following eye opening during the second postnatal week.^12^ Thus, the animal’s emotional and cognitive states, as well as sensory maturation are critical determinants of the appropriate threat responses.^2^^,^^13^ It was shown that delaying visual system maturation by dark rearing results in disrupted defensive behaviors in young mice,^14^ while conditions such as chronic stress or anxiety can induce disproportionate defensive reactions to non-threatening stimuli,^15^ potentially perturbing normal development of threat responses with long lasting repercussions. Yet how defensive behaviors develop, and whether they exhibit age and sex-specific differences in the magnitude, type of responses, and behavioral adaptation, is not fully understood.

The superior colliculus (SC), a midbrain laminar structure, is a well-established central hub for mediating defensive behaviors in numerous species, including mice and humans.^2^^,^^5^^,^^16^^,^^17^ Visual information from the retina and visual cortex is received in the superficial layers of the SC (sSC),^18^ and integrated into local circuits containing both glutamatergic and GABAergic neurons,^19^^,^^20^^,^^21^^,^^22^ which project to multiple downstream regions to compute the different behavioral outputs (e.g., freezing or escaping).^2^^,^^13^^,^^23^^,^^24^^,^^25^^,^^26^^,^^27^ For example, it was shown that SC projections to the thalamic lateral posterior (LP) nucleus and subsequently to basolateral amygdala (BLA),^24^ as well as the ventral periaqueductal gray (vPAG)^28^ are involved in mediating the freezing behavior, while the intermediate layers of the SC to dorsal periaqueductal gray (dPAG) connections mediate the escape response.^25^^,^^26^ Within these circuits, neuronal synapses form during an animal’s development^22^^,^^29^^,^^30^ and mature through life experience to enable appropriate defensive responses. Concurrently, glia cells, including astrocytes and microglia, regulate synapse formation and pruning,^31^^,^^32^^,^^33^^,^^34^ thus influencing circuit refinement and experience dependent plasticity,^35^ impacting behavior.^36^^,^^37^ However, how synapses develop during the establishment of visually evoked defensive behaviors, and the role of glia in this process, is largely unknown.

Here we use a combination of behavioral testing and immunohistochemistry (IHC) to comprehensively characterize the visually triggered innate and adaptive defensive responses as well as synaptic and glial development in wild type mice across their life span, from eye opening through adulthood. Our findings show that mice exhibit both age and sex-specific defensive behaviors, which peak in adulthood (2 months old; postnatal day (P) 60). Adolescent mice (1 month old; P30) respond to the looming stimulus by escaping to shelter (with or without freezing), while adults (2 and 4 months) of both sexes respond by freezing, yet can shift their response to escaping following increased habituation to the testing environment. Notably, mice rapidly adapt to the looming stimulus by reducing responsiveness as quickly as after 1–2 exposures, maintaining suppressed responses for up to two weeks. Adult females remain significantly more responsive than adult males, suggesting weaker behavioral adaptation. Histological analysis of synapses, astrocytes, and microglia in defensive behaviors-relevant brain regions, the superficial superior colliculus (sSC) and dorsal periaqueductal gray (dPAG)^2^ shows dynamic developmental changes in numbers of both glutamatergic and GABAergic synapses, as well as astrocytes and microglia in these brain regions, which correspond with changes in behavioral responses. Collectively, our results demonstrate that defensive response type, magnitude, and adaptation are age- and sex-dependent, and suggest the involvement of sSC and dPAG circuits in establishing these behavioral outputs in developing animals. This work provides important insight into how visually evoked threat responses are established, laying a conceptual framework for future studies focusing on the cellular mechanisms that govern the development of defensive behaviors.

## Results

**Figure 1:**
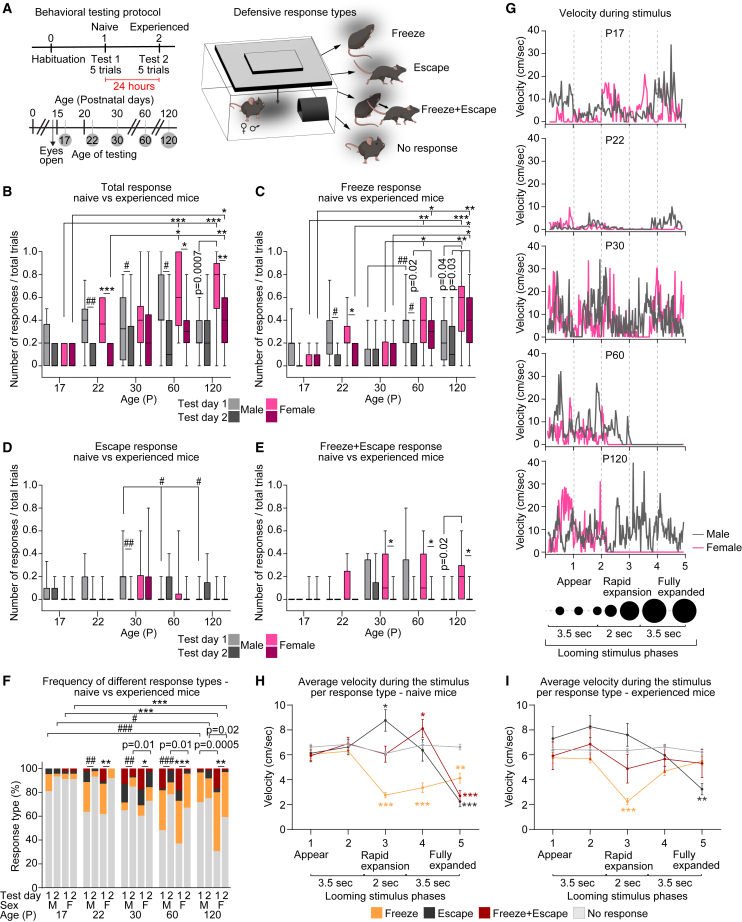
Development of defensive responses and behavioral adaptation to visually evoked looming threat (A) Top: Diagram of the behavioral experiment, including habituation and 2 testing days performed 24 h apart. Each testing day includes 5 trials of the looming stimulus. Bottom panel: schematic of the developmental time points analyzed, showing postnatal day at time of habituation. Mice are naive to the stimulus at each age on Test day 1 and retested on Test day 2 (experienced). Right panel: schematic of the testing arena. Mice are placed in the arena containing shelter; the looming stimulus is delivered via a computer monitor mounted above. Four different responses are recorded as labeled. (B–E) Average defensive responses for each type across ages, sexes, and experimental days. (B) Defensive responses (all types, total response) increase across development, peaking in adults. Responses are lower on Test day 2. (C) “Freeze” responses increase across development, peaking in adults. (D) “Escape” responses peak at P30 in males and females. Responses are increased on Test day 2 only in adult males. (E) “Freeze+Escape” responses increase across development, peaking at P30 and remaining high in older females. Responses are mostly abolished on Test day 2. Data show a box with a range, line is the median. Number of mice (N): P17M = 9, F = 9; P22M = 9, F = 10; P30M = 16, F = 14; P60M = 12, F = 14; P120M = 12 F = 13. F. Fractions of each of the defensive responses (“Freeze”—orange; “Escape”—black; “Freeze+Escape”—maroon; “No response”—gray) out of total responses are shown as percentages for each age and sex as labeled. ∗*p* ≤ 0.05, ∗∗*p* < 0.01, ∗∗∗*p* < 0.001 comparing age groups within females; ^#^*p* ≤ 0.05, ^##^*p* < 0.01, ^###^*p* < 0.001 comparing age groups within males by one-way ANOVA (B–E) or chi square goodness-of-fit test (F). Within each age, male and female comparisons were made by the Mann-Whitney test (B–E) or Fisher’s exact test (F), P value (p) on graph. G. Representative traces of video tracking data show changes in velocity during the different phases of the looming stimulus (*X* axis) in naive male (gray) and female (pink) of each age as labeled. (H–I) Changes in average velocity (both sexes combined) across the stimulus phases for each response type on Test day 1 (H) and Test day 2 (I). The duration of the “Freeze” responses (orange, e.g., decreased velocity) is shorter on Test day 2. Lines show the mean ± s.e.m of the trials for each response type across all ages and sexes. Number of responses (N): “Freeze” D1 = 122, D2 = 74; “Escape” D1 = 38, D2 = 20; “Freeze+Escape” D1 = 54, D2 = 12; “No response” D1 = 303, D2 = 419. ∗*p* ≤ 0.05, ∗∗*p* < 0.01, ∗∗∗*p* < 0.001 by two-way ANOVA comparing velocity of phase 1 to subsequent phases within each response type. Non-significant results (*p* > 0.05) are not shown (see Table S1). See also Figure S1, Table S1.

To investigate how visually triggered defensive behaviors change across development, we quantified male and female mice's responses to a visual looming threat^38^ (Figure 1A; see STAR Methods). Responses were scored (1—response, 0—no response) and further categorized into 4 types: “Freeze”—mice exhibit immobility (including alertness, attention, and prolonged freezing); “Escape”—mice move toward and enter the shelter; “Freeze+Escape”—immobility followed by movement toward and entering the shelter; “No response”—mice continue exploratory behavior such as movement or sniffing. Mice were tested at the following stages of development and maturation: postnatal days (P) 17–19; P22–24—shortly after eye opening and onset of visual experience, period of synapse development, maturation, and refinement in the visual circuits and PAG^39^^,^^40^; P30–32—adolescent, prior to onset of sexual maturity, visual circuit stabilization, and critical period of plasticity^41^; P60–62—young adult, after onset of sexual maturity; and P120–122—adult. At each age, mice are naive to the visual stimulus on Test day 1 and retested on Test day 2 (termed “experienced”) to determine adaptation (Figure 1A).

### Analysis of defensive responses and behavioral adaptation to a visual looming threat across development in male and female mice

We first compared the average responses of each sex and age group represented as a fraction of the recorded responses (regardless of response type; termed “Total response”) out of total trials per day (e.g., maximum 5 trials; Figure 1B). The responses of naive mice (Test day 1) are lowest at P17, peaking at P60 for both males (M) and females (F) (data shown as median and [range]: P17M 0.2 [0–0.5]; P60M 0.4 [0–1]; P17F 0 [0–0.2]; P60F 0.6 [0.2–1]) with no sex differences observed except for P120 females being significantly more responsive than males (P120M 0.2 [0–0.6]; P120F 0.8 [0–1]; Figure 1B). The average responses of experienced mice (Test day 2) are similarly gradually increasing with age peaking at P120, and higher in adult females than males (P17M 0 [0–0.2]; P60M 0.1 [0–0.8]; P120M 0.2 [0–0.8]; P17F 0 [0–0.2]; P60F 0.3 [0–0.8]; P120F 0.4 [0–0.8]; Figure 1B). Notably, the average responses for males are significantly lower on Test day 2 (D2) compared to Test day 1 (D1) at P22, P30, and P60, while for females it is at P22, P60 and P120 (P22M D1 0.4 [0–0.75], D2 0 [0–0.2]; P30M D1 0.33 [0–0.8], D2 0 [0–0.8]; P60M D1 0.4 [0–1], D2 0.1 [0–0.8]; P22F D1 0.37 [0.2–0.6], D2 0 [0–0.2]; P60F D1 0.6 [0.2–1], D2 0.3 [0–0.8]; P120F D1 0.8 [0–1], D2 0.4 [0–0.8]; Figure 1B), suggesting that mice adapted to the stimulus following repeated exposure at these developmental stages.

To examine responsiveness at the group level, we compared the number of responsive mice, which represented as a percentage of mice responding to at least one trial per day (Figure S1A). Naive males responded similarly across all ages tested (∼65%–90%), while females’ responsiveness is lower at P17 (44.44%), significantly increasing at later ages (∼85%–100%; Figure S1A). The percentage of male responders is reduced on Test day 2 to ∼30%–50%, statistically significant at P22 and P30 (P22M D1 88.88%, D2 33.33%; P30M D1 75%, D2 31.25%). On the other hand, the percentage of responding females is significantly decreased only at P22 (P22F D1 100%, D2 40%), while at P30–P120, female mice maintain high responsiveness of ∼60%–90% similar to naive (P30F D1 85.71%, D2 64.28%; P60F D1 100%, D2 92.85%; P120 D1 92.3%, D2 92.3%; Figure S1A), and significantly higher than males at P60 (P60M D2 92.85%, P60F D2 50%; Figure S1A). These results suggest that while repeated exposure to the looming threat reduces responses in both sexes, in males, this is caused by reduced responsiveness on both individual and group levels, while in females, this seems to be primarily caused by the reduced average response of each individual. Taken together, these data show that mice responses to the visual threat are age and sex-dependent, with young mice responding less than older mice, in line with previous work,^14^ and with adult females (P60, P120) exhibiting higher responsiveness and weaker adaptation than males.^1^

### Distinct types of defensive behaviors emerge in an age- and sex-dependent manner

Next, we asked whether male and female mice exhibit distinct response types to the looming threat at different stages of development. We separately quantified and compared the average responses and percent responders for the “Freeze,” “Escape,” and “Freeze+Escape” behaviors across the age and sex groups (Figures 1C–1E, S1B–S1D). The “Freeze” response of naive mice is highest in adults (P60 and P120) of both sexes (average responses: P17M 0.2 [0–0.5], P60M 0.4 [0–0.8], P120M 0.2 [0–0.6]; P17F 0 [0–0.2], P60F 0.4 [0–0.6], P120F 0.6 [0–1]; Figure 1C; percent of responding mice: P17 M 55.55%, P60M 83.33%, P120M 75%; P17F 22.22%, P60F 92.85%, P120F 84.61%; Figure S1B). On Test day 2, “Freeze” responses are significantly reduced in P22 mice of both sexes and P60 males (P22M D1 0.2 [0–0.75], D2 0 [0–0.2]; P60M D1 0.4 [0–0.8], D2 0 [0–0.4]; P22F D1 0.2 [0–0.6], D2 0 [0–0.2]; Figure 1C) but remain mostly unaltered in adult females (P60F D1 0.4 [0–0.6], D2 0.3 [0–0.6]; P120F D1 0.6 [0–1], D2 0.4 [0–0.8]; Figure 1C), which is significantly higher than adult males (Figure 1C). Similarly, the percentage of “Freeze” responding P60 males is reduced on Test day 2 and is significantly lower than in females, who remain highly responsive (P60M D1 83.33%, D2 33.33%; P120M D1 75%, D2 50%; P60F D1 92.85%, D2 78.57%, P120F D1 84.61%, D2 84.61%; Figure S1B).

Notably, the “Freeze” responses are significantly lower at P30 than older ages in both sexes and testing days (average responses: P30M D1 0 [0–0.4], D2 0 [0–0.4]; P30F D1 0 [0–0.4], D2 0 [0–0.4]; Figure 1C; percent responders: P30M D1 25%, D2 25%; P30F D1 35.71%, D2 42.86%; Figure S1B), suggesting that the increased total responses observed at that age (Figure 1B) are due to the mice exhibiting different behavior types. Indeed, the “Escape” response peaks at P30 in both naive male and female mice (yet it manifests earlier in males), sharply decreasing in adults (average “Escape” responses: P17M 0 [0–0.33], P22M 0 [0–4], P30M 0.2 [0–0.6], P60M 0 [0–0.2], P120M 0 [0–0.2]; P17F 0 [0–0.2], P22F 0 [0–0.2], P30F 0 [0–0.6], P60F 0 [0–0.6], P120F 0 [0–0.2]; Figure 1D; Percent responding: P17M 22.22%, P22M 33.33%, P30M 56.25%, P60M 8.33%, P120M 8.33%; P17F 11.11%, P22F 10%, P30F 42.85%, P60F 21.42%, P120F 15.38%; Figure S1C). The “Freeze+Escape” response, peaks at P60 in naive males and at P120 in females (manifesting earlier in females), and is significantly higher than males at P120 (average “Freeze+Escape” responses: P17M 0 [0–0], P22M 0 [0–0.2], P30M 0 [0–0.4], P60M 0 [0–0.8], P120M 0 [0–0.2]; P17F 0 [0–0.2], P22F 0 [0–0.4], P30F 0.1 [0–0.6], P60F 0.1 [0–0.6], P120F 0.2 [0–0.6]; Figure 1E; Percent responding: P17M 0%, P22M 11.11%, P30M 37.5%, P60M 33.33%, P120M 8.33%; P17F 11.11%, P22F 30%, P30F 50%, P60F 50%, P120F 53.84%; Figure S1D). Both these response types are strongly reduced on Test day 2 (Figures 1D, 1E, S1C and S1D) in both young (P17–22) and adult females (P60–120), unlike “Freeze” response (Figures 1C and S1B), while adult males exhibited a higher “Escape” response on Test day 2, strongly suppressing the “Freeze+Escape” response (Figures 1D, 1E, S1C, and S1D). This is further exemplified when comparing the percentage of different response types (out of the total number of responses per day) across the age and sex groups. The highest total response frequency is ∼40%–60% in both sexes (Figure 1F), which is reduced on Test day 2 to ∼10%–20% in males and young females (P17–P30) but to a lesser extent in adult females (∼30%–40% P60 and P120; Figure 1F), and showing “Freeze” as the dominant response type at all ages except P30, when “Escape” and “Freeze+Escape” dominate (>50% of all responses; Figures 1F and S1K). Overall, females were found more likely to exhibit the “Freeze+Escape” response than males (with 49 recorded responses in females versus 29 in males across all age groups and testing days), while males were less likely to respond than females (showing a higher number of “No response” for males, 426, for females, 399).

**Figure 2:**
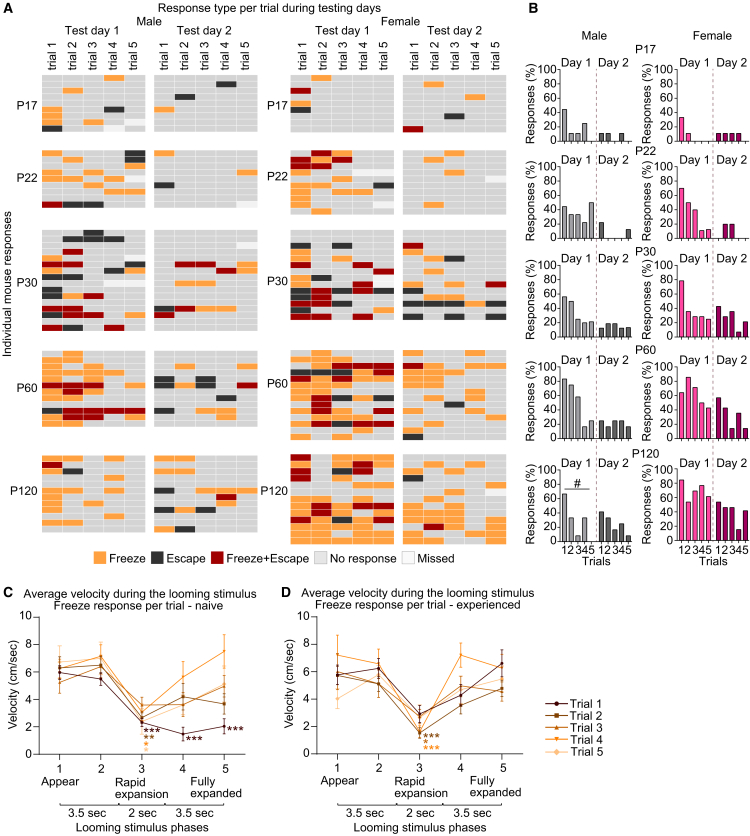
Behavioral adaptation to the looming threat is rapid (A) Heatmap of the defensive responses of each individual color-coded by response type across experimental days, developmental time points, and sex as labeled (left panel—males; right panel—females). “Freeze”—orange, “Escape”—black, “Freeze+Escape”—maroon, “No response”—gray. Missed trials (which occur if mice exceed the maximal time in the testing arena) are in white. (B) Quantification of responses shown as percent responses out of total trials per day for male (gray, left) and female (pink, right) mice. On Test day 1, most responses decrease after the first 1–2 trials, except in adult females. (C and D) Velocity of mice (both sexes combined) exhibiting the “Freeze” response as it changes during stimulus phases and trials on Test day 1 (C) and Test day 2 (D). The duration of “Freeze” responses represented, as decreased velocity, is shorter in trials 3–5 on Test day 1, while on Test day 2, “Freeze” duration is similar in all trials. Data show mean ± s.e.m. Number of responses (N): “Freeze” Trial 1 D1 = 41, D2 = 17; Trial 2 D1 = 27, D2 = 22; Trial 3 D1 = 22, D2 = 13; Trial 4 D1 = 20, D2 = 11; Trial 5 D1 = 12, D2 = 11. ∗*p* ≤ 0.05, ∗∗*p* < 0.01, ∗∗∗*p* < 0.001 by two-way ANOVA comparing the velocity of phase 1 to subsequent phases within each trial (in C and D), by chi square test (in B). Non-significant results (*p* > 0.05) are not shown (see Table S1). See also Figure S2, Table S1.

To further characterize mouse responses, we analyzed kinematic parameters, including velocity and total distance traveled (Figures S1E and S1F). We observe overall similar ambulatory abilities of the experimental groups, except P22 mice of both sexes showing lower velocity and distance than other ages (data shown as mean ± s.e.m, velocity [cm/s]: P17M D1 5.83 ± 0.45, D2 6.41 ± 0.34, P22M D1 4.52 ± 0.46, D2 5.08 ± 0.54; P120M D1 7.49 ± 0.55, D2 7.06 ± 0.47; P17F D1 6.01 ± 0.39, D2 6.7 ± 0.78, P22F D1 4.44 ± 0.33, D2 4.54 ± 0.21; P120F 6.47 ± 0.39, D2 6.42 ± 0.29; Figure S1E; Total distance [cm]: P17M D1 231.9 ± 26.14, D2 277 ± 12.15, P22M D1 176 ± 19.06, D2 210.9 ± 23.99; P120M D1 336.1 ± 26.98, D2 310.9 ± 15.7; P17F D1 267.4 ± 17.24, D2 298.6 ± 34.44, P22F D1 194.2 ± 16.38, D2 198.6 ± 11.56; P120F D1 284 ± 20.07, D2 283.6 ± 13.9; Figure S1F). To examine how mice's mobility is altered over the course of the looming stimulus, we separated the stimulus into 5 distinct phases and quantified velocities during each phase (Figures 1G and S1G–S1J; see STAR Methods). In males, velocity is significantly reduced during phases 3–5 of the stimulus (when the disc expansion occurs) only at P60 Test day 1 (shown as mean ± s.e.m [cm/s]: P60M D1 phase2 7.36 ± 0.56, phase5 4.21 ± 0.6; Figures S1G and S1H), while in females, a significant decrease in velocity is observed at ages P30–P120 on both testing days (P30F D1 phase2 7.88 ± 0.42, phase5 5.64 ± 0.56, D2 phase2 6.58 ± 0.45, phase5 4.7 ± 0.37; P60F D1 phase2 7.04 ± 0.45, phase5 4.4 ± 0.55, D2 phase2 6.72 ± 0.43, phase3 4.7 ± 0.44; P120F D1 phase2 7.71 ± 0.41, phase3 5.22 ± 0.52, D2 phase2 7.04 ± 0.37, phase3 5.2 ± 0.45; Figures S1I and S1J). This observed reduced velocity could be due to mice freezing or entering the shelter in response to the visual threat, yet since mice respond differently at each trial (see also Figure 2), averaging velocity across all trials (Figures S1G–S1J) can mask potential differences in velocities due to contrasting responses.

Thus, we sorted the velocities recorded in each trial by the manually scored response types (Figure 1A). Male and female pooled data (to increase statistical power), as well as data separated by sex, are shown in Figures 1H–1I and S1L. For responses recorded on Test day 1 (naive mice), the velocity in trials classified as “Freeze” was strongly reduced during phases 3–5 compared to phase 1 (shown as mean ± s.e.m [cm/s]: orange line, phase 1 6.03 ± 0.32, phase 3 2.75 ± 0.21, phase 4 3.35 ± 0.39, phase 5 4.12 ± 0.4; Figure 1H), suggesting increased immobility. These velocities were also significantly lower than the “No response” trials during phases 3 and 4 of the stimulus (gray line, phase 3 6.1 ± 0.19, phase 4: 6.77 ± 0.22). Noteworthy, while at an individual mouse level, the velocity during the immobility phase of the “Freeze” response is zero (see Figure 1G P60), the greater than zero value shown in Figures 1H and 1I is an average of multiple trials showing the variability in the exact timing and duration of immobility (for example, compare male and female traces, Figure 1G P60). In trials scored as “Escape,” we observed significantly increased velocity in phase 3, followed by a decrease in phase 5 compared to both phase 1 of “Escape” response trials and to the “No response” trials for each of these phases (black line, phase1 6.12 ± 0.6, phase3 8.77 ± 0.86, phase5 2.24 ± 0.43; “No response” gray line phase5 6.62 ± 0.23; Figure 1H), indicating mice rapidly moving toward the shelter and remaining there, potentially exhibiting a post-escape freezing response.^42^ Trials scored as “Freeze+Escape” were characterized by varying velocity during phases 3–4 and a significant decrease in phase 5 (maroon line, phase1 5.92 ± 0.49, phase4 8.1 ± 0.73, phase5 2.69 ± 0.44; Figure 1H). Average velocity remains constant throughout the stimulus phases in trials scored as “No response” (gray line, ∼6 cm/s; Figure 1H), as expected.

For responses recorded on Test day 2 (experienced), the velocity in trials scored as “Freeze” was significantly decreased only during phase 3 of the stimulus, increasing shortly after (orange line, phase 1 5.75 ± 0.42, phase 3 2.23 ± 0.26, phase 5 5.48 ± 0.37; Figure 1I). This suggests that mice quickly resumed exploratory behavior following initial risk-assessment response (e.g., alertness or defensive attention characterized by shorter immobility^28^ than prolonged defensive freezing), indicating adaptation. For “Escape” results, the initial velocity (phases 1–2) was higher (∼7.7 cm/s) than in naive mice (∼6 cm/s; Figure 1H), yet similarly strongly reduced in phase 5, indicating that mice began to move toward the shelter earlier in the anticipation of the looming threat (black line, phase1 7.3 ± 0.96, phase5 3.23 ± 0.45; Figure 1I). For trials scored as “Freeze+Escape,” the average velocity was mostly unchanged, unlike in naive mice. This is probably due to the low number and high variability of the recorded responses (12 responses in total), with freezing (variable durations and onset), moving, and entering the shelter occurring during different phases of the stimulus for each individual (maroon line, Figures 1I and S1L). Altogether, the kinematic data are in agreement with manual scoring, allowing further detection of additional differences in distinct types of freezing and escape behaviors, thus providing a more comprehensive understanding of how mice respond and adapt to the looming threat.

### Behavioral adaptation to the looming threat is rapid and persistent

Having observed that mice strongly reduce responsiveness to the looming threat 24 h following initial exposure (Figures 1 and S1), we next asked whether adaptation occurs on a faster timescale, such as during the five trials presented on each experimental day (Figure 1A). Analyzing the responses to each trial across age and sex groups (Figures 2A and 2B) revealed that on Test day 1, the highest number of responses (represented as percent of total responses per group) is to the first trial (∼40%–80% depending on age), decreasing to ∼10%–40% (60%–80% in P120 females) in trials 4–5 (Figure 2B). On Test day 2, response numbers to the first trial are almost identical to those recorded on the last trial of Test day 1 (for example: P60M D1 trial5 25%, D2 trial1 25%; P60F D1 trial5 42.85%, D2 trial1 57.14%; Figure 2B), confirming that mice retain the memory of behavioral adaptation 24 h later. Nevertheless, on Test day 2, the highest response numbers are still to the first trials (1–2), showing additional adaptation can occur (for example, P60F D2 trial1 57.14%, trial5 14.28%; P120M D2 trial1 41.66%, trial5 8.33%; Figure 2B). This is further reflected in the velocity of mice exhibited the “Freeze” response (Figures 2C and 2D). The prolonged immobility in phases 3–5 is mainly observed for responses recorded in trial 1 on Test day 1, with a gradual increase in mobility in later trials (shown as mean ± s.e.m [cm/s]: trial1 phase1 5.95 ± 0.5, phase3 2.32 ± 0.29, phase5 2.03 ± 0.55; trial5 phase1 6.72 ± 1.18, phase3 2.38 ± 0.92, phase5 5.12 ± 1.31; Figures 2C, S2A and S2C). On the other hand, on Test day 2, velocity changes are similar across all trials, showing decreased mobility during phase 3 only (trial1 phase1 5.76 ± 0.71, phase3 2.91 ± 0.63, phase5 6.6 ± 0.98; trial5 phase1 4.03 ± 0.72, phase3 2.64 ± 0.93, phase5 5.47 ± 0.95; Figures 2D, S2B, and S2D). Overall, the majority of naive mice responded to a total of 1–2 trials per day, with only 10 mice (out of the 118 tested) responded to all 5 trials across both testing days. Upon repeated exposure, on Test day 2, the highest number of mice are nonresponding (Figure S2E), as expected from our previous data (Figure 1F). These results demonstrate that adaptation to this stimulus occurs rapidly within 1–2 trials and persists for 24 h across all age and sex groups (except P22 males and adult females at P60 and P120). Furthermore, this shows that mice can adapt to repeated environmental stimuli not just by decreasing the number of responses, but also by reducing the magnitude or type of the response itself (e.g., switching from defensive freezing to defensive alertness/attention,^28^^,^^43^ which is characterized by reduced immobility duration), providing an important adaptive mechanism for balancing between threat response and survival behaviors.

**Figure 3:**
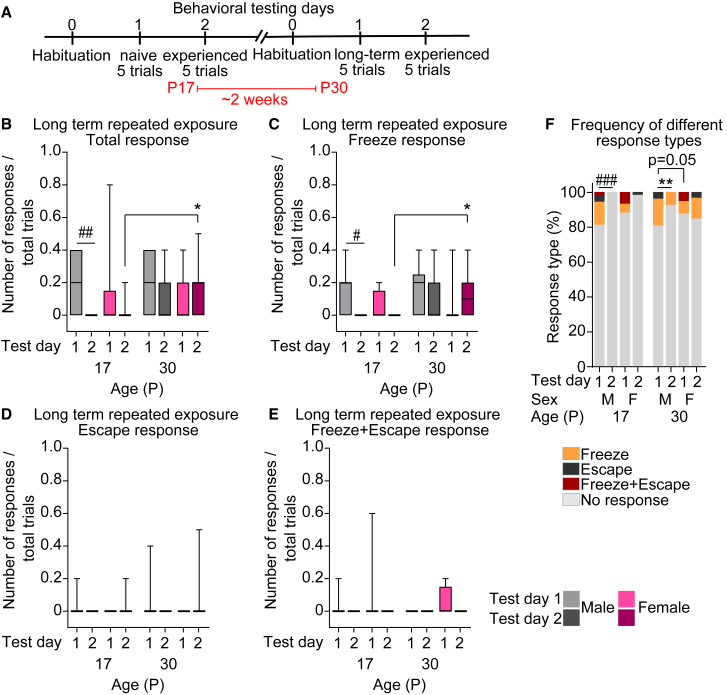
Behavioral adaptation to the looming threat is persistent (A) Diagram of the experiment: P17 mice are subjected to the behavioral test described in Figure 1A and retested 2 weeks later at P30. (B–E) Average defensive responses for each type (total response (B), “Freeze” (C), “Escape” (D), “Freeze+Escape” (E)) across ages, sex, and experimental days. Defensive responses of any type are not changed in P30 mice following initial exposure to P17 on Test day 1 but increase in females on Test day 2. Responses decrease on Test day 2, mainly in P17 male mice. Data show a box with a range, line is the median. Number of mice (N): M = 11, F = 12. (F) Fractions of each response type (“Freeze”—orange; “Escape”—black; “Freeze+Escape”—maroon; “No response”—gray) out of total responses for each age and sex, as labeled, showing mice responded to the stimulus ∼20% of the time at both ages. ∗*p* ≤ 0.05, ∗∗*p* < 0.01 comparing age groups within females by one-way ANOVA; ^#^*p* ≤ 0.05, ^##^*p* < 0.01, ^###^*p* < 0.001 comparing age groups within males by one-way ANOVA (in B–E), by chi square goodness-of-fit test (in F). Within each age, male and female comparisons were made by the Mann-Whitney test (B–E), and by Fisher’s exact test (F), P value (p) on graph. Non-significant results (*p* > 0.05) are not shown (see Table S1). See also Figure S3, Table S1.

Since we observed that adaptation is retained for 24 h, with younger mice (P17–P22) showing more robust adaptation than older mice (in particular older females), we next asked whether early life exposure to the looming stimulus has an impact on responses later in life. To test this, we subjected a separate cohort of P17 mice (Cohort 2, see STAR Methods) to the behavioral test as described in Figure 1, and retested ∼2 weeks later at P30 (Figure 3A). At P17, mice responses were low and did not differ from Cohort 1 (Figures 1 and 2) in all parameters analyzed, including average responses of naive and experienced mice (shown as median with [range]: P17M D1 0.2 [0–0.4], D2 0 [0]; P17F D1 0 [0–0.8], D2 0 [0–0.2]; Figure 3B); percentage of responders (shown as percent responding out of total group: P17M D1 63.63%, D2 0%; P17F D1 25%, D2 8.33%; Figure S3A), and types of responses displayed (Figures 3C–3E and S3B–S3D), which as shown in Figures 1 and 2, are mainly “Freeze” for males, and both “Freeze” and “Freeze+Escape” for females (shown as median and [range]: “Freeze” P17M D1 0.2 [0–0.4], D2 0 [0]; P17F D1 0 [0–0.2], D2 0 [0]; Figure 3C; percent of “Freeze” responding mice: P17M D1 54.54%, D2 0%; P17F D1 25%, D2 0%; Figure S3B; “Freeze+Escape” P17F D1 0 [0–0.6], D2 0 [0]; Figure 3E; Percent responding mice P17F D1 16%, D2 0; Figure S3D).

Remarkably, when these mice were retested 2 weeks later at P30, their responses were strongly suppressed compared to the naive P30 mice of Cohort 1 (Figures 1B and S1A) in all parameters tested including average responses (median with [range]): P30M D1 0.2 [0–0.4], D2 0 [0–0.4]; P30F D1 0 [0–0.4], D2 0.2 [0–0.5]; Figure 3B); percent responding mice (P30M D1 63.63%, D2 27.27%; P30F 41.66%, D2 58.33%; Figure S3A), and frequency of responses (∼20%; Figure 3F compared to ∼40% in Cohort 1 mice; Figure 1F). The “Escape” and “Freeze+Escape” responses were almost completely abolished, as evident from average responses (“Escape” response: P30M D1 0 [0–0.4], D2 0 [0]; P30F D1 0 [0], D2 0 [0–0.5]; Figure 3D; “Freeze+Escape” response P30M D1 0 [0], D2 0 [0]; P30F D1 0 [0–0.2], D2 0 [0]; Figure 3) and percent responders (“Escape” response: P30M D1 9%, D2 0%; P30F D1 0%, D2 8.33%; Figure S3C; “Freeze+Escape” response: P30M D1 0%, D2 0%; P30F D1 25%, D2 0%), in contrast to these strongly upregulated response types in naive P30 mice (Figures 1D, 1E, S1C, and S1D). Of the recorded responses at P30, the most common response for males was “Freeze,” while females responded with “Freeze” and “Freeze+Escape” (Figure S3E). These data suggest that early life exposure to a stimulus that does not elicit a strong threat response can nevertheless leave a long-term memory impacting responsiveness in later life stages.

### Increased habituation shifts defensive response types in adult mice

**Figure 4:**
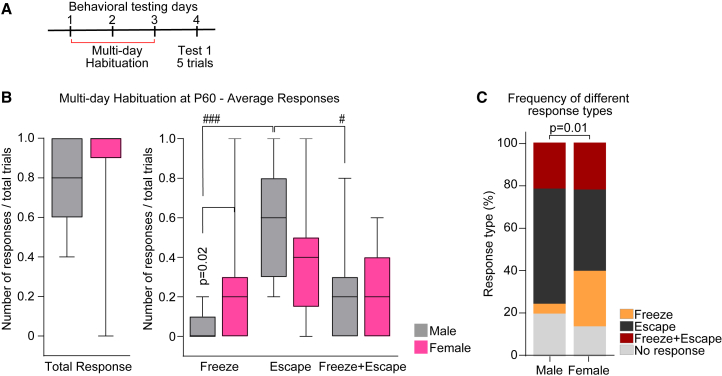
Increased habituation shifts defensive response types in adult mice (A) Diagram of experiment: mice are subjected to three consecutive days of habituation in the testing arena with shelter and tested as in Figure 1A on the fourth day. (B) Average defensive responses for each type as labeled in males (gray) and females (pink). Total responses on the left, different response types (“Freeze,” “Escape,” and “Freeze+Escape”) on the right. “Escape” responses are highest on both sexes, while in females, “Freeze” responses are higher than in males. Data show a box with a range; the line is the median. Number of mice (N): M = 13, F = 10. (C) Fractions of each response (“Freeze”—orange; “Escape”—black; “Freeze+Escape”—maroon; “No response”—gray) out of total responses for each sex as labeled, showing “Escape” as the dominant response in both sexes. ^#^*p* ≤ 0.05, ^###^*p* < 0.001 comparing age groups within males by one-way ANOVA (in B). Male and female comparison by Mann-Whitney test (B), Fisher’s exact test (C); P on graph. Non-significant results (*p* > 0.05) are not shown (see Table S1). See also Figure S4, Table S1.

Our data show that “Freeze” is the most common response to the looming threat in all conditions tested (except for naive P30 mice). It was shown that mice escape to shelter more frequently when familiarity with their environment is increased, such as through repeated habituation.^9^^,^^13^ Thus, we asked if strengthening the mice's familiarization with the testing arena and shelter location through increased habituation could influence the behavioral response. To test this, male and female P60 mice were habituated for 3 consecutive days (Cohort 3, see STAR Methods; Figure 4A), and tested on the fourth day as in Figure 1, Test day 1.

Under these conditions, the overall average response is higher than in mice tested after 1 habituation day (shown as median and [range]: P60M 0.8 [0.4–1]; P60F 1 [0–1]; Figure 4B; compare to P60M 0.4 [0–1]; P60F 0.6 [0.2–1]; Figure 1B), with percent responders of 90%–100% (Figure S4A) and response frequency of ∼80% (Figure 4C). Moreover, there is a shift in behavior showing significantly higher “Escape” responses (statistically significant in males; Figure 4B) in contrast to P60 mice tested after 1 habituation day (Cohort 1; Figure 1; see also Table S1 and Figure 4). Though similar in both sexes, this phenomenon is more prominent in males, whose “Freeze” response is almost completely abolished upon increased habituation (average responses shown as median and [range]: “Freeze” P60M 0 [0–0.2], P60F 0.2 [0–1]; “Escape” P60M 0.6 [0.2–1], P60F 0.4 [0–1]; “Freeze+Escape” P60M 0.2 [0–0.8], P60F 0.2 [0–0.6]; Figure 4B; percent responding mice: “Freeze” P60M 23.07%, P60F 70%; “Escape” P60M 100%, P60F 80%; “Freeze+Escape” P60M 69.23%, P60F 60%; Figure S4A), showing “Escape” as the dominant response type (P60M 67.3% Figure S4B), significantly different than response type frequency in females (P60F “Escape” frequency 44.18%), and strongly resembling the frequency and magnitude of the responses exhibited by the adolescent mice (P30; Figures 1D and S1C). These results demonstrate that familiarization with the environment can influence the defensive response strategy, supporting previous work.^9^^,^^13^ This also suggests that younger mice (P30) form quicker or stronger memories than older mice (P60) as they escaped to shelter after a short habituation time, suggesting that developmental changes in circuit maturation and activity, such as experience dependent plasticity, may contribute to these behavioral differences.

Taken together, our data show that mice exhibit age- and sex-specific defensive behaviors in response to a visual threat, which can be modified through habituation and repeated exposure.

### Developmental analysis of synapses and glia in defensive behavior-relevant brain regions, the superficial superior colliculus and dorsal periaqueductal gray

**Figure 5:**
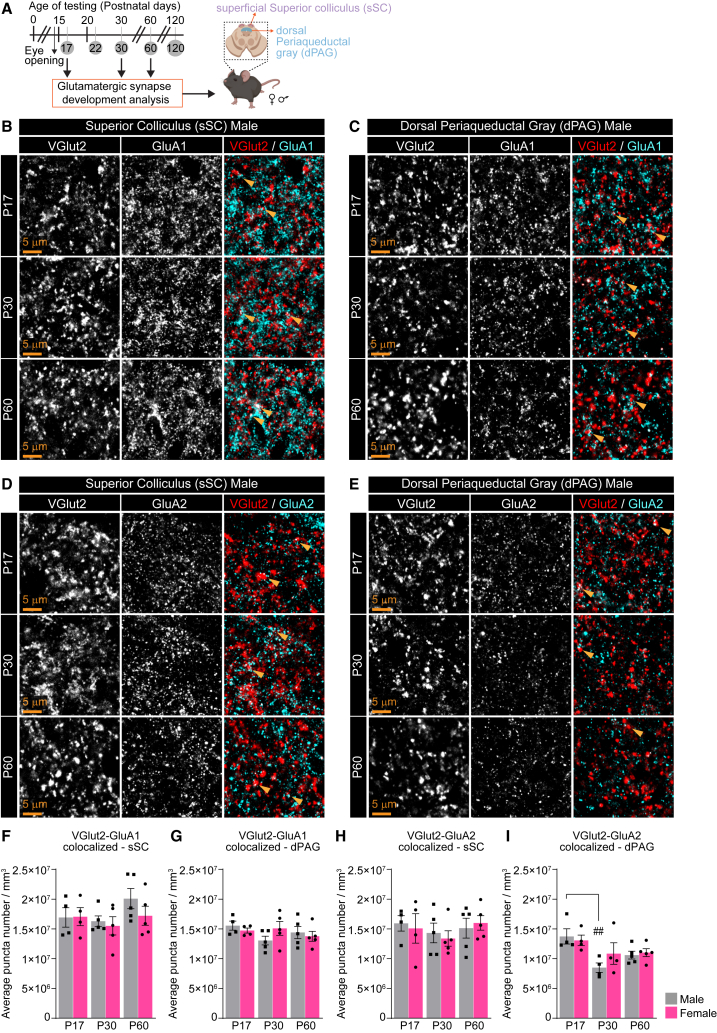
Developmental analysis of glutamatergic synapses in the sSC and dPAG (A) Schematic of experiment: Brain tissue is collected from male and female mice at P17, P30, and P60; IHC to quantify synapse types as indicated is performed in the superficial superior colliculus (sSC) and dorsal periaqueductal gray (dPAG). (B, C, F, G) Immature (GluA1-containing) synapses are unaltered across the developmental timepoints in either sSC (B, F) or dPAG (C, G). Example images of the presynaptic VGlut2, postsynaptic GluA1, and merged (synapses) in each age and brain region, as labeled in male mice (B–C), and quantification of synapse number per mm^3^ represented as colocalization between VGlut2 and GluA1 for both sexes (F–G) are shown. (D, E, H, I) Mature (GluA2-containing) synapse numbers are unchanged in the sSC across development in both sexes (D, H), but are developmentally decreased in the dPAG (E, I). Example images of VGlut2, GluA2, and merged (synapses) in each age and brain region in male mice (D-E) and quantification of synapse number per mm^3^ for both sexes (H and I) are shown. Plots show mean ± s.e.m. Squares and circles indicate the average of signal for each individual mouse. Number of mice/sex group (N): P17 *N* = 4; P30, P60 *N* = 5. Scale bars = 5 μm. Arrowheads mark representative colocalized puncta. ^##^*p* < 0.01 comparing age groups within males by one-way ANOVA. Non-significant results (*p* > 0.05) are not shown (see Table S1). See also Figure S5, Table S1.

**Figure 6:**
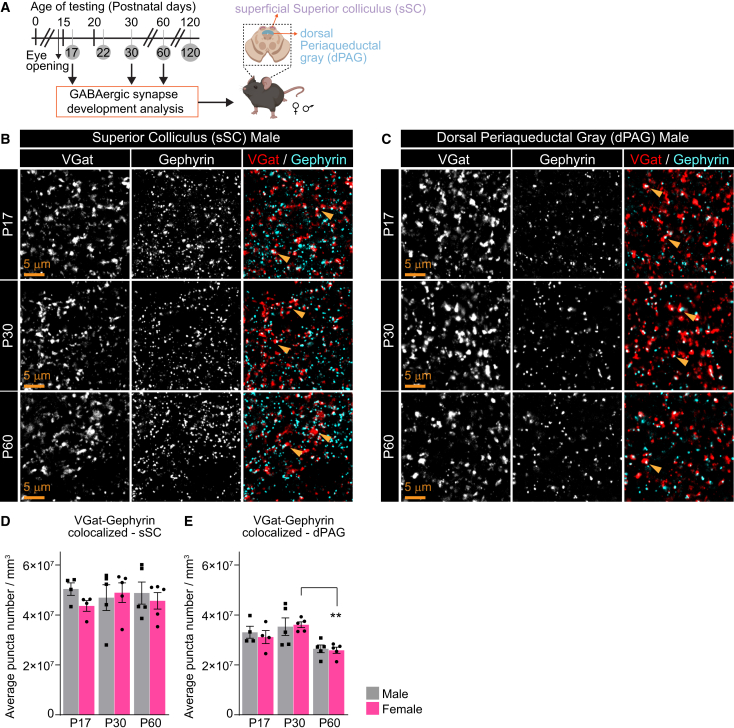
Developmental analysis of GABAergic synapses in the sSC and dPAG (A) Schematic of experiment: Brain tissue is collected from male and female mice at P17, P30, and P60; IHC to quantify synapse types as indicated in the superficial superior colliculus (sSC) and dorsal periaqueductal gray (dPAG). (B and C) Example images of the presynaptic VGat, postsynaptic Gephyrin, and merged (synapses) in each age and brain region as labeled in male mice. (D and E) Quantification of synapses in puncta number per mm^3^ shows a colocalization between VGat and Gephyrin for both sexes in the sSC (D) and dPAG (E) is shown. The number of GABAergic synapses in the sSC is unaltered across the developmental time points but is decreased in adults (P60) compared to young mice (P17) in the dPAG. Plots show mean ± s.e.m. Squares and circles indicate the average signal for each individual mouse. Number of mice/sex group (N): P17 *N* = 4; P30, P60 *N* = 5. Scale bars = 5 μm. Arrowheads mark representative colocalized puncta. ∗∗*p* < 0.01 comparing age groups within females by one-way ANOVA. Non-significant results (*p* > 0.05) are not shown (see Table S1). See also Figure S6, Table S1.

**Figure 7:**
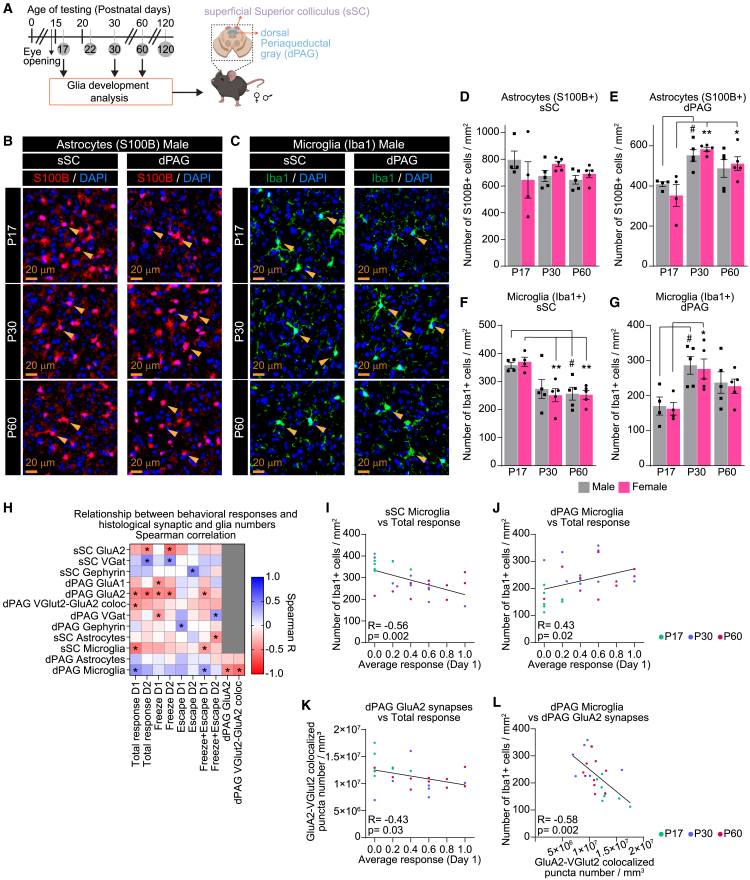
Developmental analysis of astrocytes and microglia in the sSC and dPAG and correlation between cellular and behavioral parameters (A) Schematic of experiment: Brain tissue is collected from male and female mice at P17, P30, and P60; IHC analysis is performed to quantify cell types as indicated in the superficial superior colliculus (sSC) and dorsal periaqueductal gray (dPAG). (B and C) Example images of the astrocytes labeled by a specific marker S100B (B) and microglia labeled by Iba1 (C) in each age and brain region, as labeled in male mice. (D and E) Quantification of astrocytes represented as the number of S100B positive cells per mm^2^ for both sexes in the sSC (D) and dPAG (E). No change in astrocyte numbers in the sSC, while astrocyte numbers are increased at P30 in the dPAG. (F and G) Quantification of microglia represented as the number of Iba1 positive cells per mm^2^ for both sexes in the sSC (F) and dPAG (G). Microglia numbers are developmentally decreased in the sSC but increase in the dPAG. No sex differences are observed. Plots show mean ± s.e.m. Squares and circles indicate the average signal for each individual mouse. Number of mice/sex group (N): P17 *N* = 4; P30, P60 *N* = 5. Scale bars = 20 μm. Arrowheads mark representative S100B or Iba1 positive cells. ∗*p* ≤ 0.05, ∗∗*p* < 0.01 comparing age groups within females; ^#^*p* ≤ 0.05 comparing age groups within males by one-way ANOVA. Non-significant results (*p* > 0.05) are not shown (see Table S1). (H–L) Spearman correlation analysis compares histological data with behavioral responses for individual mice. (H) Heatmap shows Spearman R values for the different pairwise comparisons as labeled. Negative correlations are in red, and positive correlations are in blue. An asterisk inside each square indicates a statistically significant correlation (*p* ≤ 0.05). (I and J) Correlation plots for pairwise comparisons as labeled show a significant negative correlation between behavioral response (total response) and microglia in the sSC (I), a positive correlation between behavioral response and microglia in the dPAG (J), a negative correlation between behavioral response and GluA2-containing synapses in the dPAG (K), and a negative correlation between dPAG GluA2-containing synapses and dPAG microglia (L). For each comparison, Spearman R and *p* values are shown within each plot. Datapoints are color-coded by age: P17 (green), P30 (blue), and P60 (pink). *N* = 25–27 mice. See also Figure S7, Table S1.

At the cellular level, behavioral outputs are mediated through synaptic communication within neuronal circuits. In addition, glia cells, in particular astrocytes and microglia, contact synapses and modulate multiple aspects of synapse development and pruning, thus contributing to circuit refinement and influencing behavior.^31^ Yet, how synapses and glia are developing within the brain regions responsible for the visually triggered defensive behaviors is unknown. To understand the cellular mechanisms driving the age- and sex-dependent defensive behaviors, we used immunohistochemistry (IHC) on mouse brain sections to characterize the changes in the numbers of structural excitatory (glutamatergic) and inhibitory (GABAergic) synapses, astrocytes, and microglia at timepoints when the main developmental behavioral changes were observed: P17, P30, and P60 (Figures 1 and 2). We focused on the superficial superior colliculus (sSC), which receives visual input from the retina^18^ and is needed to detect the visual threat, and the dorsal periaqueductal gray (dPAG), which integrates multiple inputs, including from the SC, and has been implicated in the escape (“Escape”) response^25^ (Figures 5, 6, and 7). To quantify glutamatergic synapses, we immunostained sections for the vesicular glutamate transporter 2 (VGlut2), to mark the presynaptic terminals of retinal ganglion neurons in the sSC,^44^ and glutamatergic inputs from the SC in the dPAG.^25^ To detect the postsynaptic sites, we costained sections for two subunits of AMPA type glutamate receptors, GluA1, which marks immature synapses as well as excitatory synapses on GABAergic neurons, and GluA2, which marks mature synapses, to determine circuit maturation states^39^ (Figure 5 and S5). To quantify inhibitory synapses, we co-immunostained for the presynaptic vesicular GABA transporter (VGat) and inhibitory postsynaptic density marker Gephyrin^45^ (Figures 6 and S6). For each section, the number of pre- and postsynaptic puncta, as well as the colocalization between the two signals indicating a synaptic contact, was quantified and compared between groups. To analyze changes in glia populations during these developmental stages, we stained for S100*β* (S100B) to mark astrocytes and Iba1 to mark microglia in both brain regions (Figures 7 and S7).

#### Mature excitatory glutamatergic synapses are developmentally regulated in the dorsal periaqueductal gray

Analysis of GluA1-containing synapses (Figure 5A) revealed no difference in levels of either pre- or postsynaptic proteins (Figures 5B, 5C, S5A, S5B, S5E–S5H) or colocalized puncta (VGlut2-GluA1 colocalized; Figures 5B–5C, 5F, 5G, S5A, and S5B) across age and sex in either brain region. The mature GluA2-containing synapse numbers remain constant across age and sex groups in the sSC (Figures 5D and 5H), but are decreased at P30 compared to P17 in the dPAG by ∼20%–40% in both sexes, statistically significant in males (VGlut2-GluA2 colocalized; P17M 1.37E+07 ± 1.27E+06, P17F 1.31E+07 ± 8.73E+05; P30M 8.50E+06 ± 7.96E+05, P30F 1.09E+07 ± 1.80E+06; P60M 1.06E+07 ± 6.57E+05, P60F 1.10E+07 ± 6.65E+05; Figures 5E, 5I, and S5D). These differences are mainly driven by the developmental decrease in the postsynaptic GluA2 levels (P17M 1.31E+08 ± 3.42E+06, P17F 1.29E+08 ± 3.90E+06; P30M 1.16E+08 ± 4.26E+06, P30F 1.21E+08 ± 4.71E+06; P60M 1.12E+08 ± 2.17E+06, P60F 1.12E+08 ± 1.32E+06; Figures 5E, S5D, and S5L), with no change in the presynaptic VGlut2 puncta number (Figure S5K). Similarly, GluA2 levels are developmentally decreased in the sSC and statistically significant in females, with no change in VGlut2 (P17M 1.44E+08 ± 4.77E+06, P17F 1.5E+08 ± 5.38E+06; P30M 1.3E+08 ± 7.48E+06, P30F 1.35E+08 ± 4.26E+06; P60M 1.36E+08 ± 5.03E+06, P60F 1.29E+08 ± 6.09E+06; Figures 5D, S5C, S5I, and S5J). These results show that glutamatergic synapses undergo developmental regulation in the sSC and dPAG, with GluA1-containing immature synapses remaining relatively constant across these ages in both brain regions, while GluA2-containing mature synapses are downregulated in the dPAG at P30, likely due to synaptic pruning and subsequent circuit refinement.^46^

#### Inhibitory GABAergic synapses are differentially developmentally regulated in the superficial superior colliculus and dorsal periaqueductal gray

Next, we characterized the developmental changes in GABAergic inhibitory synapses by quantifying the levels of the presynaptic protein VGat and postsynaptic Gephyrin (Figure 6A). In the sSC, the numbers of inhibitory synapses remain constant across all age and sex groups with no sex differences, though an increase (∼19%) in VGat levels at P30 compared to P17, statistically significant in females, was observed (P17M 5.5E+07 ± 2.01E+06, P17F 5E+07 ± 2.6E+06; P30M 6.2E+07 ± 2.4E+06, P30F 6E+07 ± 2.9E+06; Figures 6B, 6D, S6A, S6C, and S6D). Conversely, inhibitory synapse number in the dPAG is decreased in adults (P60) compared to younger ages in both sex groups by ∼30%, statistically significant in females (P17M 3.31E+07 ± 2.39E+06, P17F 3.11E+07 ± 2.62E+06; P30M 3.53E+07 ± 3.51E+06, P30F 3.61E+07 ± 1.15E+06; P60M 2.64E+07 ± 1.53E+06, P60F 2.59E+07 ± 1.24E+06; Figures 6C, 6E, S6B). These changes are driven by the downregulation of the presynaptic VGat protein levels (P17M 5.65E+07 2.38E+06, P17F 5.53E+07 1.95E+06; P30M 5.83E+07 2.17E+06, P30F 5.67E+07 2.18E+06; P60M 4.56E+07 2.63E+06, P60F 4.66E+07 2.38E+06; Figure S6E), with no change in Gephyrin puncta in either brain region (Figure S6F). These results demonstrate that inhibitory circuits are structurally modulated in adolescence in the sSC and in adulthood in the dPAG.

#### Astrocytes and microglia are differentially regulated during development in superficial superior colliculus and dorsal periaqueductal gray

A decrease in synaptic numbers can be explained by synapse pruning, a developmental process executed by astrocytes and microglia.^47^ Yet the prevalence of microglia and astrocytes in brain regions relevant to defensive behaviors during these developmental stages is unknown. We quantified astrocytes labeled by S100β (S100B) and microglia labeled by Iba1 in the sSC and dPAG across the age and sex groups as above (Figure 7A). There are no sex differences in the development of microglia and astrocytes in either brain region (Figures 7B–7G, S7A, and S7B). The number of astrocytes in the sSC is constant across all age and sex groups (Figures 7B, 7D, and S7A), yet significantly increases in dPAG at P30–60 by ∼60% compared to P17 (shown as mean ± s.e.m [cell number per mm^2^]: P17M 408.4 ± 12.08, P17F 352.2 ± 54.73; P30M 552.9 ± 29.34, P30F 582.9 ± 10.37; P60M 487.7 ± 44.45, P60F 511.3 ± 34.38; Figures 7B, 7E, and S7A).

For microglia, we observed differential developmental modulation in these brain regions. While in the sSC there is a significant decrease in Iba1 positive microglia numbers at P30–60 by ∼30% compared to P17 (P17M 357.8 ± 10.81, P17F 370.4 ± 16.44; P30M 273.7 ± 33.32, P30F 251.4 ± 23.26; P60M 256.7 ± 22.72, P60F 252.8 ± 15.28; Figures 7C, 7F, and S7B), in the dPAG, microglia numbers are significantly increased by ∼60% at P30 compared to P17 (P17M 170.2 ± 25.6, P17F 162.4 ± 17.83; P30M 286.4 ± 25.35, P30F 275.8 ± 28.49; P60M 237.4 ± 30.49, P60F 226.8 ± 20.29; Figures 7C, 7G, S7B). Total cell numbers marked by the nuclear marker DAPI are relatively unchanged across age and sex groups with a small but significant decrease in the sSC at P30–60 by ∼15% compared to P17 (P17M 2020 ± 38.35, P17F 1,834 ± 116.2; P30M 1,827 ± 46.87, P30F 1,936 ± 67.57; P60 1,718 ± 43.34, P60F 1,740 ± 30.23; Figures S7C and S7D). Overall, astrocytes make up ∼30% of all cells in the sSC and ∼20–30% in the dPAG, while microglia are ∼10%–17% of total cell numbers in both regions. These data align well with previously published analysis in the developing visual cortex.^39^

Altogether, these histological studies demonstrate the dynamic developmental modulation in both excitatory and inhibitory circuits, astrocytes, and microglia in two key brain regions involved in the detection and execution of visually triggered defensive behaviors.

### Developmental changes in behavioral responses correlate with synaptic and glial levels in the superficial superior colliculus and dorsal periaqueductal gray

To examine the relationship between the observed cellular and behavioral changes, we performed a correlation analysis using Spearman correlation on mice for whom both the behavioral and histological data were collected. This analysis revealed significant correlations between several behavioral response types and cellular parameters (Figures 7H and S7E). For example, the magnitude of the response (total response) was significantly negatively correlated with microglia in the sSC (Figure 7I; *R* = −0.56, *p* = 0.002) and positively correlated with microglia in the dPAG (Figure 7J; *R* = 0.43, *p* = 0.02), as expected based on the differential developmental changes in microglia numbers within these regions. In addition, we find a significant negative correlation between GluA2 levels in both brain regions with multiple behavioral parameters (such as “Total response” and “Freeze” response), suggesting a potential cellular mechanism for how GluA2-containing synapses are refined to enable the development of these behaviors (Figure 7H and 7K; *R* = −0.43, *p* = 0.03). This is further supported by a significant negative correlation between microglia in the dPAG and GluA2 levels, suggesting microglia involvement in refining these circuits (Figure 7L; *R* = −0.58, *p* = 0.002). We also observed a significant differential correlation between inhibitory synaptic proteins in the dPAG and the “Freeze” (negative correlation, Figure S7F; *R* = −0.38, *p* = 0.04) and “Escape” behaviors (positive correlation, Figure S7G; *R* = 0.41, *p* = 0.03), suggesting the involvement of inhibitory circuits in the developmental switch from freezing to escaping that we observed (Figure 1). Interestingly, some synaptic proteins in the sSC significantly correlated with responses recorded on Test day 2 (experienced mice) but not with Test day 1 (naive), suggesting a potential involvement of these circuits in the observed behavioral adaptation (Figure 7H).

To summarize, this analysis reveals a potential direct association between the cellular and synaptic changes and the observed developmental behavioral responses, providing clues to the underlying cellular mechanisms that drive these behaviors.

## Discussion

With this work, we investigate how defensive responses to a visual threat are manifested across the life span from eye opening through adulthood and analyze the changes in structural synapses and glia within the relevant brain regions. The main findings are:

• Mice responses to a novel visual threat increase with age, peaking in adulthood (P60).
• Mice exhibit different types of defensive behaviors in an age and sex dependent manner. Adolescent mice (P30) respond by escaping or freezing followed by escaping, with the latter response being more prominent in female mice.
• Adult mice respond by freezing, while increasing familiarization to the environment (by increasing habituation) shifts the response to escaping, more prominently in males.
• Behavioral adaptation to the looming threat is rapid (following 1–2 exposures) and is retained for up to two weeks. Behavioral adaptation is more robust in young mice (P17–22) than in adults of both sexes and in adult males compared to adult females.
• Histological analysis shows dynamic developmental changes in structural glutamatergic and GABAergic synapse numbers, astrocytes, and microglia in the dPAG and sSC, which correspond with behavioral changes.

### Defensive behavioral responses to a novel visual threat increase with age

The ability of animals to detect a threatening stimulus and elicit a defensive response is dependent on their ability to process sensory information. In addition to the sensory component, animals must learn to discern between threatening and innocuous stimuli and adapt their responses accordingly. In mice, visual circuits mature after eye opening during the second postnatal week,^39^^,^^48^^,^^49^^,^^50^ enabling visually triggered threat responses to develop. We demonstrate that shortly after the eye opening (P17), both male and female mice respond weakly to a novel looming stimulus, with responses gradually increasing in maturing animals, peaking in adulthood (P60; Figure 1), in line with previous work.^14^ At 4 months old (P120), female mice are significantly more responsive than males, suggesting potential sex differences in threat responses^1^ at older adult (P120), but not young adult (P60) stages.^13^^,^^51^ A possible explanation for the lack of responsiveness in young mice could be due to immature visual or motor functions. We found similar locomotor abilities in all the tested age groups (Figure S1), indicating motor function cannot explain the differences in responses. Furthermore, it was shown that although mice’ eyes continue to grow through adolescence, their visual responses are stabilized at around P16,^52^ suggesting that the low responses at P17 are also not due to lack of vision. Alternatively, young mice may not recognize the stimulus as threatening and ignore it.^53^ Indeed, our data show that young mice quickly adapt to the looming stimulus, suppressing their responses after 1–2 trials and retaining the memory of this behavioral adaptation through adolescence (Figure 5, Figure 6, Figure 7 and Figure 5, Figure 6, Figure 7), thus confirming their ability to perceive the stimulus at P17, without it triggering a defensive behavior. On the other hand, the increasing responses of older mice likely stem from learning through life experience to associate a novel looming stimulus with danger.^54^ This elicits an initial response; however, they quickly adapt to repeated stimulation, thereby reducing their responsiveness.

Importantly, varying the conditions of the visual stimuli, such as changing the direction of movement^5^ or contrast of the looming object against the background^4^ can impact the magnitude and type of defensive responses observed in adult animals. It was shown that defensive responses are enhanced when auditory and visual stimuli are combined compared to the response to each individual stimulus,^55^ yet how these different parameters affect developing animals has not been addressed. Furthermore, whether similar developmental changes in defensive behaviors occur in response to other sensory stimuli, such as auditory or tactile, is unknown. Future studies investigating how different sensory stimuli impact defensive responses in developing animals and how changes to the stimulus parameters modify these behaviors are needed to address this knowledge gap.

### Distinct types of defensive responses are age and sex dependent and can be modified by increasing familiarization with the environment

Freezing and/or escaping are the most commonly observed defensive behaviors to a visual threat in mice,^2^^,^^5^^,^^13^^,^^25^ yet how they are manifested in maturing animals of both sexes has not been thoroughly investigated. We quantified three distinct types of responses: “Freeze”—mice became immobile during the stimulus; “Escape”—mice escaped to the shelter; “Freeze+Escape”—immobility followed by movement into the shelter (Figure 1), which we find to be age- and sex-specific. The “Escape” response was observed mainly in young (P17, P22) males and adolescent males and females (P30), sharply decreasing in adulthood, while “Freeze+Escape” was mainly observed in young females (P22), peaking in adolescence (for both sexes) and stabilizing in adulthood. “Freeze” response was common to both sexes and showed a bimodal developmental profile, being high at P22 and P60, but dipping at P30 when “Escape” and “Freeze+Escape” responses dominate. These results fit well with previous work showing peak escape response at P30;^14^ however, unlike our findings, mice also exhibited escape behaviors also in adulthood, while freezing responses were not strongly affected by age.^14^ Others have reported robust escape behaviors in adult mice^10^^,^^13^^,^^51^ suggesting experimental conditions, such as stimulus type or habituation, could explain these discrepancies. Indeed, increasing the habituation of adult mice to 3 days (instead of 1 day) shifted the behavior from freezing to escaping (Figure 4), suggesting familiarization with the environment plays a key role in behavioral manifestation. Interestingly, adolescent mice (P30) readily escape to the shelter, potentially indicating stronger memory. This is likely due to enhanced plasticity of brain circuits occurring at this age,^46^^,^^56^ while our data show altered numbers in synapses and glia at P30 in the dPAG, known to be involved in the escape response,^25^ and significant correlation between behavioral responses and several synaptic and glia components (specifically GluA2 and microglia; Figure 5, Figure 6, Figure 7 and Figure 5, Figure 6, Figure 7) in this region. It is currently unknown if increasing habituation in younger mice would elicit a similar behavioral switch and whether perturbing the correct development of dPAG circuits will have an impact on these behaviors. Future work will determine the mechanistic link between the function of these circuits and specific defensive behaviors in adolescent mice.

### Behavioral adaptation to the looming stimulus is rapid and persistent, but sex dependent only in adults

Animals must balance their threat responsiveness with survival behaviors (such as food foraging or sleep); thus, behavioral adaptation to repeated environmental stimuli is critical to ensure normal function. It was shown that male mice habituate upon repeated exposure to the looming stimulus by decreasing responses;^7^ however, how adaptation occurs at different developmental stages in each sex and its duration was largely unknown. Our data shows that mice adapt to repeated stimulus exposure by reducing responsiveness following 1–2 exposures and retain suppressed responses for up to 24 h or 2 weeks when first exposed at P17. Adaptation was observed at all developmental stages on both the group level, showing decreased number of responding mice (percent responding), and the individual level, showing decreased number of responses (average response) as well as the magnitude of the response, such as shorter immobility during the “Freeze” response, which could indicate a different defensive strategy such as defensive alertness or attention^28^^,^^43^ (Figures 1 and 2). “Freeze” is the most common adaptive response (on Test day 2; Figures 1F and S1K), especially in females, except for P30, while males also respond by escaping. With this, adaptation to repeated stimulus exposure in males resembles the behavioral switch observed upon increased habituation to the environment, when male mice strongly suppressed freezing but increased escape responses. These data further support the conclusion that mice respond by increasing alertness and displaying brief immobility when the potential threat is not imminent,^2^^,^^57^ allowing them to resume normal function. Notably, animals exhibit a wide range of risk assessment behaviors, including alertness, defensive attention, as well as prolonged defensive freezing (including threat anticipatory freezing and defensive immobility),^28^ all of which can manifest as pausing of exploratory behavior and eliciting short- or longer-term immobility. While these types of behavior were not distinguished by the manual scoring criteria of the “Freeze” response used in this study, our kinematic analysis showed differences in the duration of immobility (for example, between Test day 1 and Test day 2 responses) suggests that mice exhibit distinct types of freezing behavior when naive to the looming stimulus versus following repeated exposure. Future work will require careful characterization of distinct types of freezing responses, combining manual observations with kinematics data to identify age- sex- and adaptation-specific types of freezing behaviors.

Behavioral adaptation is similar in both sexes at young ages (P17–30); however, adult male mice show more robust adaptation than females (P60), as evident by significantly lower “Freeze” responses and percent responders on Test day 2 (Figure S1). Notably, although “Freeze” responses are higher in females, both sexes show similar immobility time (Figure S2), indicating that adaptation still occurs in females but to a lesser extent. Importantly, no sex differences in the average responses of naive P60 mice were observed, suggesting that female mice may persist in a higher state of alertness or stress, thus continuing to perceive the stimulus as threatening. Since these differences are not observed in young animals (prior to the onset of sexual maturity), it would be important to determine the role of sexual maturation and the females' estrous cycle and circulating hormone levels in driving these divergent responses. It was shown that female rats in late diestrus exhibited stronger hypoxia triggered panic-like behavior than during other stages of the estrous cycle,^58^ while a growing body of work demonstrates the effects of estradiol on neuronal activity^59^ and astrocyte-neuron communication,^60^ suggesting a potential role in regulating the females' defensive responses. More work is needed to better understand the sex-specific processes that govern the adaptation of defensive responses in adults.

### Development of escape behaviors corresponds with changes in structural synapses and glia in the superficial superior colliculus and dorsal periaqueductal gray

Defensive behaviors involve complex coordination of neuronal circuits across multiple brain regions.^2^ Within these circuits, the correct establishment of neuronal synaptic connections is critical for generating the proper behavioral outputs. Yet how synapses develop and are regulated in these regions is largely unknown. To understand the cellular mechanisms driving the development of visually evoked defensive behaviors, we characterized age and sex-dependent changes in the numbers of structural glutamatergic and GABAergic synapses, astrocytes, and microglia. We also conducted a correlation analysis to examine the potential functional relationship between these cellular and behavioral metrics. In this study, we focused on two regions known to play a critical role in detecting, processing, and eliciting the defensive responses, yet where synaptic and glial development have not been investigated: the superficial superior colliculus (sSC), which receives visual input from the retina needed for sensory processing, and the dorsal periaqueductal gray (dPAG), which receives input from the SC and other brain regions to mediate the escape response.

In the sSC, the number of structural synapses did not change from P17 through adulthood (P60; Figure 5, Figure 6, Figure 7 and Figure 5, Figure 6, Figure 7), though a small but significant reduction in GluA2 levels and an increase in VGat across the developmental stages were observed. This suggests that visual input relevant circuits are stabilized earlier than the visual cortex, where synapses undergo pruning and refinement during the third postnatal week, stabilizing toward P30.^39^ This is further supported by the observed decrease in microglia and no change in astrocyte numbers at P30 compared to P17 (Figure 7), which are known to modulate synapses during development. It is possible that synapse development and circuit refinement occur earlier in development in the sSC.^22^^,^^29^ Indeed, the number of VGlut2-GluA1 synapses at P6 observed in our previous work (∼7.9E+06 synapses/mm^3^; calculated from raw data)^44^ is lower than at P17 obtained in this study (∼1.7E+07), providing further support for this conclusion. Future studies will determine how structural synapses and glia in the sSC change across postnatal development by analyzing earlier timepoints, including P3, P7, and P14, as well as investigate how perturbing synapse development by manipulating synaptic proteins or glia function, such as VGat, GluA2, and microglia, which are significantly correlated with behavioral outputs (Figure 5, Figure 6, Figure 7H and S7E), impacts defensive responses later in life. It will also be important to examine potential developmental changes in synaptic function, such as synapse strength and plasticity, which are not readily inferred from structural data.

We observed dynamic developmental changes in structural synapse numbers, synaptic protein levels, and glia in the dPAG. Specifically, GluA2-containing mature synapses are significantly decreased at P30 compared to P17, while GABAergic inhibitory synapse numbers are significantly decreased at P60 (Figure 5, Figure 6, Figure 7 and Figure 5, Figure 6, Figure 7), suggesting a similar developmental trajectory to the visual cortex^39^ for circuit refinement. These structural changes may explain the increased escape behavior at P30 and its disappearance at P60. Furthermore, we observed a significant increase in both astrocyte and microglia numbers in dPAG at P30, suggesting a potential mechanism underlying decreased synapses due to glial pruning (Figure 7). Indeed, our correlation analysis identified a potential link between the magnitude of behavioral response, GluA2, and microglia levels and showed differential correlation between the “Freeze” and “Escape” response types and the inhibitory proteins VGat and Gephyrin (Figure 5, Figure 6, Figure 7H–7L and S7E–S7G). These correlative observations provide an important conceptual framework for future experiments. For example, blocking microglia^61^ or astrocyte^32^ phagocytic ability in the dPAG will enable us to examine the impact of synaptic pruning on the development of escape responses. Furthermore, since we observed that adult mice can elicit the escape response upon additional habituation, it would be important to analyze how synaptic function and plasticity are impacted by varying habituation days.

We did not observe sex differences in either synapse or glia development within the regions studied here, contrary to other brain regions such as the cortex and hippocampus.^62^^,^^63^^,^^64^^,^^65^ This is not surprising since both sex groups similarly detected the visual threat and elicited the escape response, suggesting the involvement of additional brain regions in the observed sex-specific behavioral outputs (e.g., different types of responses and adaptation). Future work focusing on regions such as the amygdala will determine the precise sexual dimorphic circuits that control the development of defensive responses.

To summarize, this work demonstrates that defensive behaviors to a visual threat are manifested differently depending on the animal’s developmental stage and sex, and can be modified upon changing environmental conditions. Furthermore, mice show rapid and prolonged adaptation to repeated exposure, with adult females exhibiting weaker adaptation than males. We also observe correlative synaptic and glial developmental changes in the sSC and dPAG, suggesting a potential cellular mechanism for how escape behaviors develop. By understanding the age and sex-specific types of defensive behaviors, this study enables future investigations aimed at determining the role of distinct brain regions, synaptic types, and glia in the development and maintenance of visually evoked threat responses.

### Limitations of the study

This work identifies the age and sex-specific innate and adaptive defensive behaviors and suggests correlative synaptic and glial changes within the dPAG and sSC as mediators of these responses. Nevertheless, multiple additional brain regions are known to be involved in defensive behaviors, including those related to mediating distinct response types and/or sex-dimorphic behaviors, such as the amygdala, thalamic nuclei, or ventral PAG, which were not analyzed here. In addition, our histological analysis focuses on structural changes and thus can miss potential functional impacts of synaptic or glial activity on these behaviors. Accordingly, while our correlative studies identified interesting relationships between cellular and behavioral changes, they are limited as they do not provide a direct functional link or causation. Future work leveraging genetic or functional perturbations to the brain regions identified here, incorporation of functional assays such as calcium imaging, electrophysiology, and fiber photometry, as well as analysis of additional brain regions, will facilitate further functional dissection of the development of defensive responses and identify the roles of specific synaptic and glial populations in these behaviors.

## Resource availability

### Lead contact

Requests for further information and resources should be directed to and will be fulfilled by the lead contact Isabella Farhy-Tselnicker (ifarhy@bio.tamu.edu).

### Materials availability

This study did not generate new unique reagents.

### Data and code availability

• Microscopy and behavioral recording data reported in this article will be shared with the lead contact upon request.
• This article does not report the original code.
• Any additional information required to reanalyze the data reported in this article is available from the lead contact upon request.

## Acknowledgments

We thank Dr. Chen Farhy for helping with R scripts and Dr. Heath Blackmon for advice on statistical analysis. We thank Gillian Imrie, Madison B. Gray, Ridita Ray Basunia, and all members of the Farhy lab for the critical evaluation of the article. Some figure components were designed using BioRender. This work was funded by 10.13039/100000002NIH R01NS133047 to I.F.-T. and NS133047 to J.M.

## Author contributions

G.L.A. investigation, formal analysis, validation, writing original draft, and supervision; M.M. investigation; J.M. investigation, supervision, formal analysis, and validation. R.R. investigation and formal analysis. D.M. formal analysis, visualization, data curation, and supervision; R.C.F. data curation and formal analysis; V.R. formal analysis and validation; I.F-T. conceptualization, funding acquisition, formal analysis, supervision, project administration, visualization, writing-original draft, and writing review and editing.

## Declaration of interests

The authors declare no competing interests.

## Declaration of generative AI and AI-assisted technologies in the writing process

During the preparation of this work, the authors used Google Gemini in order to correct grammar, spelling and suggest synonyms or phrasing. After using this tool, the authors reviewed and edited the content as needed and take full responsibility for the content of the publication.

## STAR★Methods

### Key resources table

**Table:** 

REAGENT or RESOURCE	SOURCE	IDENTIFIER
**Antibodies**		

Guinea pig anti VGlut2	Millipore	Cat#AB2251; RRID:AB_2665454
Rabbit anti GluA1	Millipore	Cat#AB1504; RRID:AB_2113602
Rabbit anti GluA2	Millipore	Cat#AB1768I; RRID:AB_2922404
Guinea pig anti VGat	Synaptic systems	Cat#131004; RRID:AB_887873
Rabbit anti Gephyrin	Synaptic systems	Cat#147008; RRID:AB_2619834
Guinea pig anti S100B	Synaptic systems	Cat#287004; RRID:AB_2620025
Rabbit anti Iba1	Waco	Cat#016-20001; RRID:AB_839506

**Chemicals, peptides, and recombinant proteins**		

Ketamine	Comparative medicine program, Texas A&M University	
Xylazine	Comparative medicine program, Texas A&M University	

**Experimental models: Organisms/strains**		

Mice	Jackson labs	Cat#000664; RRID:IMSR_JAX:000664

**Software and algorithms**		

ImageJ (FIJI)	Open source	RRID:SCR_003070
Imaris	Bitplane	RRID:SCR_007370

**Other**		

Superfrost plus slides	Fisher	Cat#1255015
Leica Thunder Imager	Leica	RRID:SCR_026034
Zeiss LSM 900 Confocal microscope	Zeiss	RRID:SCR_022263
Cryostat CM1950	Leica	RRID:SCR_018061

### Experimental model and study participant details

All animal work was approved by the Texas A&M University Institutional Animal Care and Use Committee (Animal Use Protocol #2023-0244).

#### Mice

Wild type mice *C57Bl6/J* were purchased from Jackson Labs (Jax #000664) and bred in-house for experiments. Mice were maintained in the Texas A&M University ILSB animal facility, under a 12-hour light:dark cycle with *ad libitum* access to food and water. Both male and female mice were used at the following ages: postnatal days (P) 17-19, 22-24, 30-32, 60-62, and 120-122. All mice are weaned at the age of P21 except for age group P22-24, who were kept in the parental cage until the end of behavioral testing (P24).

For behavioral testing (see below), to minimize subject variability due to handling and life experience differences, mice were minimally handled by experimenters, and only mice born and raised in the animal facility of the laboratory were used for the behavioral testing. Mice were rigorously examined to ensure testing is performed at correct developmental stages, including monitoring for eye opening, body size, and fur appearance according to the Jackson labs mouse developmental chart.^66^ Male and female mice were tested separately except at ages P17 and P22 (pre-weaned) who remained with parents as mixed sex litters.

All behavioral testing and tissue collection for histology were performed between 1-6 pm.

### Method details

#### Behavioral testing

*Behavioral test setup* was in accordance with Daviu et al. (2020)^38^ with some modifications to enable testing of mice at young ages. The testing setup consists of a rectangular arena (17W X 40L X 20H cm) with a plastic hut (11 cm in diameter, 5 cm height) placed in one corner of the cage serving as shelter for mice to escape to. The hut used in this work is identical to that used in the mouse home cages to facilitate quicker familiarization and habituation to the testing environment. A computer monitor is mounted 40 cm above the arena floor, connected to a computer through which the looming stimulus is delivered. A video camera is placed above the cage to record mouse movement during the stimulus for offline analysis.

*The looming stimulus* consisted of a 9 second video showing an expanding black disc (2-20cm in diameter) on a white background to mimic a looming threat (file available from Daviu et al., (2020)^38^). The stimulus begins when a black disc (2 cm) appears on the computer screen (∼3.5 seconds), rapidly expands to 20 cm diameter (∼2 seconds) and remains fully expanded for ∼3.5 seconds (see Figure 1G). This stimulus has been thoroughly characterized in previous studies to elicit robust defensive responses in mice.^13^^,^^38^

##### Behavioral testing protocols (visual looming-shadow task description)

Cohort 1: Analyzing defensive responses during developmental stages (Figures 1, 2, S1, and S2). The test was administered over 3 consecutive days and consisted of a habituation day (day 0), followed by 2 consecutive testing days (Test day 1, Test day 2; Figure 1A). The short habituation (1 day) is necessary to allow testing of young mice at specific postnatal days (e.g., P17, P22). During habituation, mice are placed in the testing arena one at a time, and allowed to explore for 13 minutes, without the looming stimulus played. On each Testing day, mice are placed in the arena and subjected to 5 trials of the looming stimulus. The first stimulus is played after a minimum of 3 minutes of free exploration, with a minimum of 1 minute interval between subsequent stimuli, during which time mice resume exploratory behavior. Mice are removed from the arena after receiving 5 trials, spending a maximum of 16 minutes in the arena and returned to home cages even if they did not receive 5 trials. Stimulus is played only if a mouse is outside the shelter and engaged in exploratory behavior. Test day 2 is identical to Test day 1, occurring ∼24 hours later. Mice are returned to the vivarium between testing days.

Cohort 2: Early life exposure and long-term memory analysis (Figure 5, Figure 6, Figure 7 and S3). Mice at P17 were subjected to the protocol identical to Cohort 1. After Test day 2, mice were returned to their home cages, weaned at P21 and maintained in the vivarium until P30. Then, mice were retested using same protocol as Cohort 1.

Cohort 3: Analyzing the impact of increased habituation on response types in adult mice (Figure 5, Figure 6, Figure 7 and S4). The test was administered to mice at P60 over 4 consecutive days and consisted of 3 habituation days, and one testing day. The habituation and testing days are as in Cohort 1.

##### Scoring of defensive behavioral responses

The behavioral responses to the looming stimulus are analyzed in two ways:

1 Manual scoring - by an experimenter either live during the testing, or offline from recorded videos. For each stimulus, 4 types of responses are recorded: “Freeze” – was used to describe periods of immobility during the looming stimulus if a mouse’s snout and body is immobile for a minimum of 1 second, thus including alertness, defensive attention and defensive freezing behavioral characteristics (e.g., stretch-attend posture); “Escape” – the mouse is moving towards and entering the shelter; “Freeze+Escape” – a period of immobility followed by movement and entering the shelter; “No response” – mouse continues exploratory behavior or movement with no directed change due to the stimulus.
2 Kinematics analysis – calculating velocity and distance traveled during the stimulus from recorded videos. Mouse movement during the stimulus was tracked using ImageJ (NIH) manual tracker plugin (https://imagej.net/ij/plugins/track/track.html) to obtain XY coordinates. All videos were tracked only during the stimulus (9 seconds), with the mouse’s nose/ head used as source for tracking. The XY coordinates obtained were used to calculate the distance and velocity during stimulus using the linear motion equation: Distance = $\sqrt{{\left(x2-x1\right)}^{2}+{\left(y2-y1\right)}^{2}}$; Velocity = $\frac{\sqrt{{\left(x2-x1\right)}^{2}+{\left(y2-y1\right)}^{2}}}{\left(t2-t1\right)}$; t2-t1 = time interval between frames. To quantify the change in velocity during the course of the stimulus, we subdivided the 9-second stimulus into 5 phases, ∼1.8 seconds long (20% of the total video time) which correspond to different stages of the black disc appearance: Phase 1 - a small black disc appears on the monitor; Phase 2 - black disc remains small; Phase 3 - rapid expansion of the black disc; Phase 4 - black disc expansion reaches maximal size; Phase 5 - fully expanded disc remains displayed on the monitor until disappearance (Figure 1G).

##### Exclusion criteria

1) Mice who missed more than 2 stimuli due to, for example remaining inside the shelter, on either testing days, were removed from the study.
2) Videos in which the mice are out of frame for more than 10 frames at a time were not tracked and removed from the dataset.

#### Mouse tissue collection

Tissue was collected at the following developmental time points: post-natal day (P) 19, 24, 32, 62, 122, following the behavioral testing (These ages are labeled as P17, P30, and P60 in the Figures and text to indicate “age groups” for consistency).

Mice were anaesthetized by I.P. injection of 100 mg/kg Ketamine /20 mg/kg Xylazine mix (obtained from Comparative Medicine Program at Texas A&M University) and transcardially perfused with PBS, then 4% PFA at room temp. Brains were removed and incubated in 4% PFA overnight at 4C, then washed 3 X 5 min with PBS, and cryoprotected in 30% sucrose for 2-3 days, before being embedded in TFM media (VWR #100496-345), frozen in dry ice-ethanol slurry solution, and stored at -80C until use. Brains were sectioned using a cryostat (Leica CM1950) in sagittal or coronal orientations depending on experimental needs at a slice thickness of 18-20 μm. Sections were mounted on Superfrost plus slides (Fisher #1255015) and either used immediately for histological procedures (IHC [Immunohistochemistry]) or stored at -80C for later use. Three to five mice from each sex and age group were used. For each mouse, 2-3 sections were imaged and analyzed.

#### Immunohistochemistry in mouse brain tissue

IHC was performed as described.^39^ Briefly, slides containing the brain sections were blocked for 1h at room temp in blocking buffer containing antibody buffer (100 mM L-lysine (Millipore-Sigma #L5501-25G) and 0.3% Triton X-100 (Millipore-Sigma #T9284-100ML) in PBS) supplemented with 10% heat-inactivated normal goat serum (Gibco #16210072). Primary antibodies diluted in antibody buffer with 5% goat serum were incubated overnight at 4C. The next day slides were washed 3 X 5 min with PBS with 0.2% Triton X-100 and secondary antibodies conjugated to Alexa fluor were applied for 2h at room temp. Slides were mounted with the SlowFade Gold with DAPI mounting media (Life Tech #S36939), covered with #1.5 glass coverslip (Fisher #152450) and sealed with clear nail polish. The following antibodies were used: Gp anti VGlut2 (Millipore #AB2251, 1:1000), Rb anti GluA1 (Millipore #AB1504, 1:400), Rb anti GluA2 (Millipore #AB1768-I, 1:2500), Gp anti VGat (Synaptic systems #131004, 1:250), Rb anti Gephyrin (Synaptic systems #147008, 1:500), Gp anti S100*β* (Synaptic systems, #287004 1:200), Rb anti Iba1 (Wako #016-20001, 1:300). All secondary antibodies were applied at 1:500 dilution.

#### Imaging and analysis

*Fluorescent Microscopy* was performed to image the developmental changes in numbers of astrocytes and microglia (Figures 7 and S7) using a Leica THUNDER Imager with LED3 light source, and sCMOS camera (Leica DFC9000) at 10X or 20X magnification. Single plane images (2048 X 2048 pixels) containing the superficial layers of the superior colliculus (sSC), and dorsal periaqueductal gray (dPAG) brain regions were acquired for each section separately. Thunder processing (Leica LASX software) was performed using default parameters for single plane imaging (instant computational clearing) to increase resolution and image clarity in the same way for all images.

*Confocal Microscopy* was performed to image synaptic proteins for synapse number analysis (Figures 5, 6, S5, and S6) using Zeiss LSM 900 confocal microscope at 63X magnification. For each section, 1024 X 1024 pixels (101.4 X 101.4 X 2.79 μm) thick z stack image was obtained (pixel size 0.09 X 0.09 X 0.31 μm; 10 slices per 2.79 μm stack) from sSC, or dPAG.

In each experiment, a pair of male and female mice was compared at each age, slides were sectioned, immunostained and imaged on the same day using set exposure.

*Image analysis* was done with ImageJ (NIH) or Imaris (Bitplane) software as described:

*Quantification of astrocyte and microglia numbers* (Figures 7 and S7): Thunder processed images labeled with anti S100*β* (S100B) antibody (to mark astrocytes) and Iba1 (to mark microglia) were analyzed using semi-automatic custom-made macro in ImageJ (adapted from^39^). For each image, sSC and dPAG regions were manually cropped and saved to a new file. For each of the different signals (S100B, Iba1 or DAPI – nuclear marker), images were thresholded in the same way for each section and the “Analyze Particles” function was used with a size range of 20-500 pixels to automatically separate and quantify the numbers of S100B or Iba1 positive cells as well as total cell number (DAPI positive). Resulting cell counts were normalized to the total area of analyzed image to obtain the number of cells per square millimeter.

*Quantification of pre and postsynaptic puncta, and synapses* (Figures 5, 6, S5, and S6) was performed as previously described.^39^^,^^44^ 3D z stack images were analyzed using Imaris software (Bitplane). Positive puncta of GluA1, GluA2, VGlut2, VGat or Gephyrin were selected by size and intensity by thresholding the images in the same way for each section. Then, colocalization between each 2 pre-postsynaptic pairs (VGlut2-GluA1, VGlut2-GluA2, VGat-Gephyrin) was calculated. Puncta were considered colocalized if the distance between them was ≤ 0.5 μm. The number of colocalized puncta was obtained and compared between the experimental groups. Two to three sections per mouse were imaged for each sex, age, and brain region, and the experiment was repeated in 4-5 male and female pairs. Puncta numbers are represented as normalized to the total volume of analyzed image in cubical millimeter. Example images show a single z plane from the same location in the stack for both sexes.

### Quantification and statistical analysis

All data is presented as either median with range, mean ± standard error of mean (S.E.M), or percentages, with single data points representing the data obtained for each individual mouse/ trial, as indicated in each figure legend. Statistical analysis was performed using Prism software (Graphpad) or using R or Python. Where applicable, data was tested for normal distribution using Shapiro-Wilk test, to determine the appropriate statistical test to be performed. Pairwise comparisons were done by t-test (normal distribution) or Mann-Whitney Rank Sum test (non-normal distribution), while multiple group comparisons were done using one-way Analysis of Variance (ANOVA) with post hoc Tukey, Dunn’s or Dunnet’s tests (normal distribution), or Kruskal-Wallis ANOVA on ranks with post hoc Tukey test (non-normal distribution). Fisher’s exact test (with Monte Carlo simulation when contingency table was larger than 2X2) was used to determine whether there was a significant difference in the distribution of responses across categories (Figures S1A–S1D, S3A–S3D, and S4A), while a Chi-Square Goodness-of-Fit Test was conducted to identify the most frequent response (Monte Carlo simulation was performed if average of observations was <5; Figures 1F, S1K, 3F, S3E, 4C, and S4B). Two-way ANOVA with post-hoc Tukey test was performed to determine the contribution of response type and stimulus phase to changes in velocity (Figures 1H, 1I, S1L; 2C, 2D, and S2A–S2D). P value ≤ 0.05 is considered statistically significant. The sample sizes, statistical tests, and significance are shown in each Figure and Figure legend. The complete statistical analysis including tests used and statistical values in addition to P values are listed in Table S1 for each figure panel as labeled.

## Supplemental information

Document S1. Figures S1–S7

Table S1. Data and statistical analysis, related to Figures 1–7 and S1–S7 Table containing a comprehensive summary of all data and statistical analysis tests performed. The data is organized into separate tabs corresponding to each Figure as labeled.
