# Supplementary material for: Developmental analysis of visually evoked defensive behavior identifies age and sex-specific responses and underlying synaptic and glia changes
Source: iScience. 2025 Nov 11;28(12):113997. doi: 10.1016/j.isci.2025.113997 (PMC12723169; doi:10.1016/j.isci.2025.113997)
Supplement: Document S1. Figures S1–S7 [file mmc1.pdf]

iScience, Volume *28*

Supplemental information

Developmental analysis of visually evoked defensive behavior identifies age and sex-specific responses and underlying synaptic and glia changes

Georgia Lee Albrecht, Rebekah Ramirez, Delaram Moradpour, Jordan Mar, Rafael Colla Fortes, Matthew A. McGregor, Vishnuvasan Raghuraman, and Isabella Farhy-Tselnicker

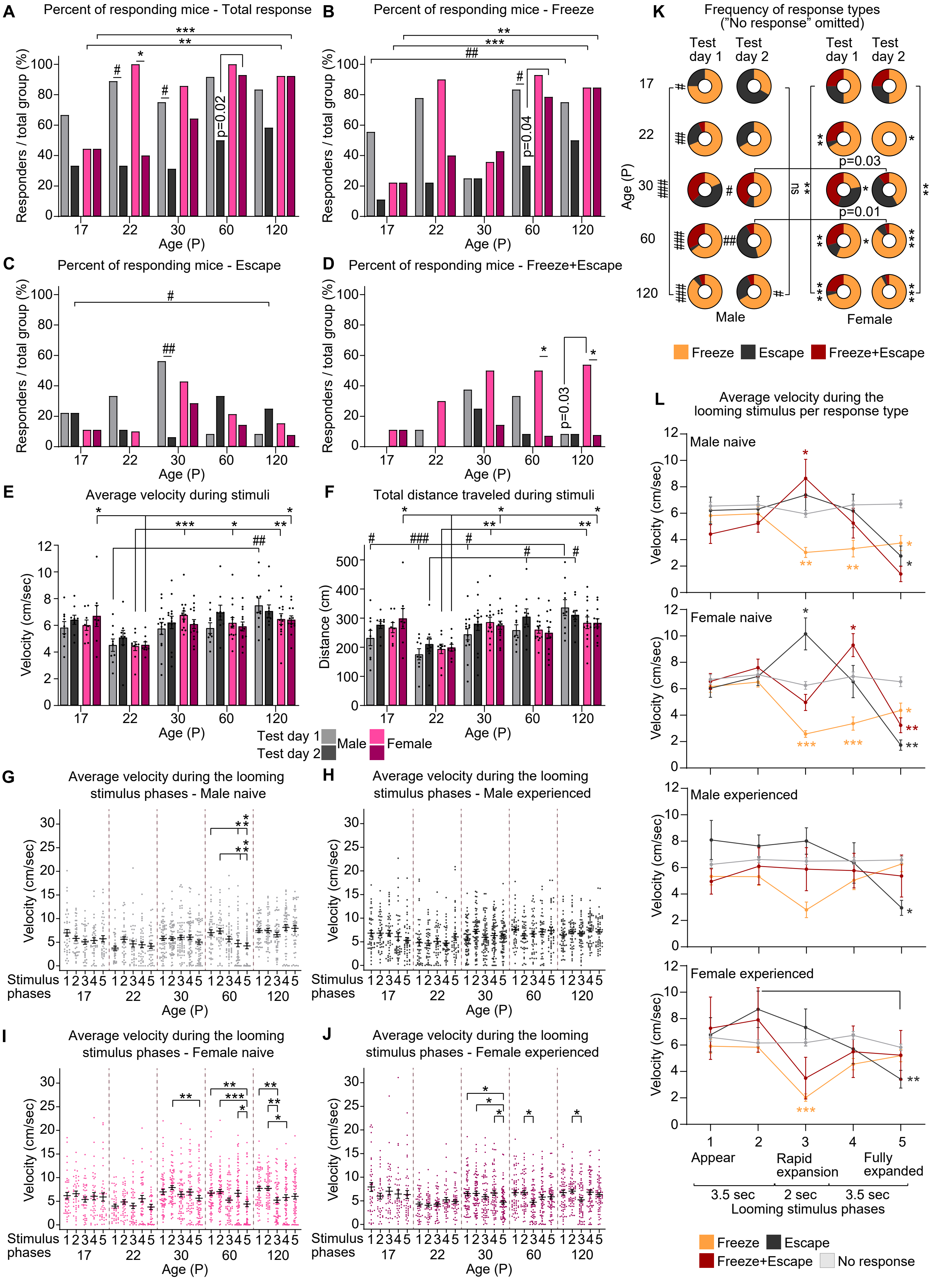
A
Percent of responding mice - Total response
Responders / total group (%)
Age (P)
B
Percent of responding mice - Freeze
C
Percent of responding mice - Escape
D
Percent of responding mice - Freeze+Escape
E
Average velocity during stimuli
Velocity (cm/sec)
F
Total distance traveled during stimuli
Distance (cm)
Test day 1
Test day 2
Male
Female
G
Average velocity during the looming stimulus phases - Male naive
H
Average velocity during the looming stimulus phases - Male experienced
I
Average velocity during the looming stimulus phases - Female naive
J
Average velocity during the looming stimulus phases - Female experienced
Stimulus phases
K
Frequency of response types
("No response" omitted)
Test day 1
Test day 2
Age (P)
Male
Female
Freeze
Escape
Freeze+Escape
L
Average velocity during the looming stimulus per response type
Male naive
Female naive
Male experienced
Female experienced
Velocity (cm/sec)
Appear
Rapid expansion
Fully expanded
3.5 sec
2 sec
3.5 sec
Looming stimulus phases
No response

Figure S1. Development of defensive responses and behavioral adaptation to visually evoked looming threat, Related to Figure 1. See also Table S1. **A-D.** Number of responders for each behavior represented as percentage of mice responding to at least one trial per day out of the total group number. **A.** Percent responding males is similar across development and is reduced on Test day 2. The present of responding females increases after P17 and is unchanged on Test day 2 in adults. **B.** Percent responders by “Freeze” is decreased at P30 in both sexes. **C.** Percent responders by “Escape” peaks at P30 in males and females. **D.** Percent responders by “Freeze+Escape” peaks at P30 and remains high in older females. Graphs show percentages, see Fig. 1 for N. **E-F.** Velocity (E) and total distance traveled (F) averaged across the entire 9 seconds of the stimulus shows similar kinematic parameters for male and female mice. Velocity and distance are lower at P22 than other ages in both sexes. Data shows mean ± s.e.m. Data points represent the average velocity for each mouse. Number of mice (N): P17 M=9, F=8; P22 M=9, F=9; P30 M=14, F=14; P60 M=9, F=13; P120 M=10 F=13. **G-J.** Velocity changes during stimulus phases in naive (G, I) and experienced (H, J) mice as labeled. Data points show average velocity for each individual trial, lines are mean ± s.e.m. **K.** Doughnut plots showing the fractions of different response types from Fig. 1F, with the “No response” omitted. In **A-K**, $^{*}p \leq 0.05$, $^{**}p < 0.01$, $^{***}p < 0.001$ comparing age groups within females per test day; $^{\#}p \leq 0.05$, $^{\#\#}p < 0.01$, $^{\#\#\#}p < 0.001$ comparing age groups within males per test day by Fisher’s exact test (A-D), one-way ANOVA (E-J), or Chi square Goodness-of-fit test (K). Within each age and testing day, male and female comparison by t-test or Fisher’s exact test (K), P value (*p*) on graph. Non-significant results ($p > 0.05$) are not shown (see Table S1). **L.** Changes in average velocity during stimulus phases for each response type as in Fig. 1H-I separated by sex as labeled. Lines show an average of the trials for each response type. Number of responses (N): Male: “Freeze” D1=47, D2=20; “Escape” D1=19, D2=8; “Freeze+Escape” D1=16, D2=7; “No response” D1=157, D2=213. Female: “Freeze” D1=75, D2=54; “Escape” D1=19, D2=12; “Freeze+Escape” D1=38, D2=5; “No response” D1=146, D2=206. $^{*}p \leq 0.05$, $^{**}p < 0.01$, $^{***}p < 0.001$ by two-way ANOVA comparing velocity of phase 1 to subsequent phases within each response type. Non-significant results ($p > 0.05$) are not shown (see Table S1).

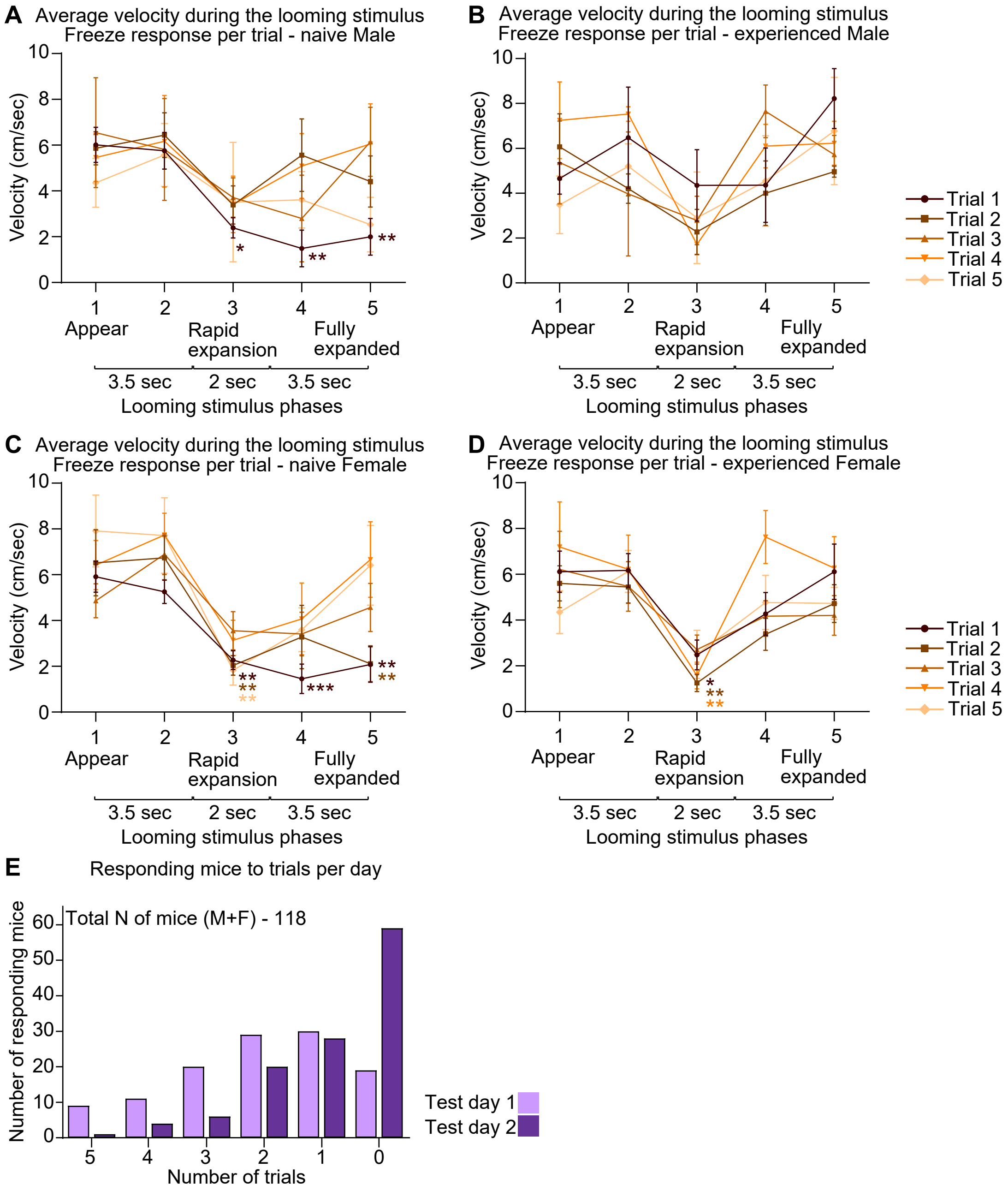
A
Average velocity during the looming stimulus
Freeze response per trial - naive Male
B
Average velocity during the looming stimulus
Freeze response per trial - experienced Male
C
Average velocity during the looming stimulus
Freeze response per trial - naive Female
D
Average velocity during the looming stimulus
Freeze response per trial - experienced Female
Velocity (cm/sec)
Appear
Rapid expansion
Fully expanded
3.5 sec
2 sec
3.5 sec
Looming stimulus phases
Trial 1
Trial 2
Trial 3
Trial 4
Trial 5
E
Responding mice to trials per day
Total N of mice (M+F) - 118
Number of responding mice
Number of trials
Test day 1
Test day 2

Figure S2. Behavioral adaptation to the looming threat is rapid, Related to Figure 2. See also Table S1. **A-D.** Velocity of mice exhibiting the “Freeze” response as it changes during stimulus phases plotted by the different trials on each test day in males (A-B) and females (C-D). Data shows mean ± s.e.m. Number of responses (N): Males: Trial 1 D1=19, D2=4; Trial 2 D1=11, D2=6; Trial 3 D1=6, D2=3; Trial 4 D1=7, D2=3; Trial 5 D1=4, D2=4. Females: Trial 1 D1=22, D2=13; Trial 2 D1=16, D2=16; Trial 3 D1=16, D2=10; Trial 4 D1=13, D2=8; Trial 5 D1=8, D2=7. *$p \leq 0.05$, **$p < 0.01$, ***$p < 0.001$ by two-way ANOVA comparing velocity of phase 1 to subsequent phases within each trial. Non-significant results ($p > 0.05$) are not shown (see Table S1). **E.** Numbers of mice responding to the different number of trials on each testing day as labeled. The majority of mice responded to 1-2 trials per day on Test day 1 (light purple), while on Test day 2 – the majority of mice did not respond (dark purple).

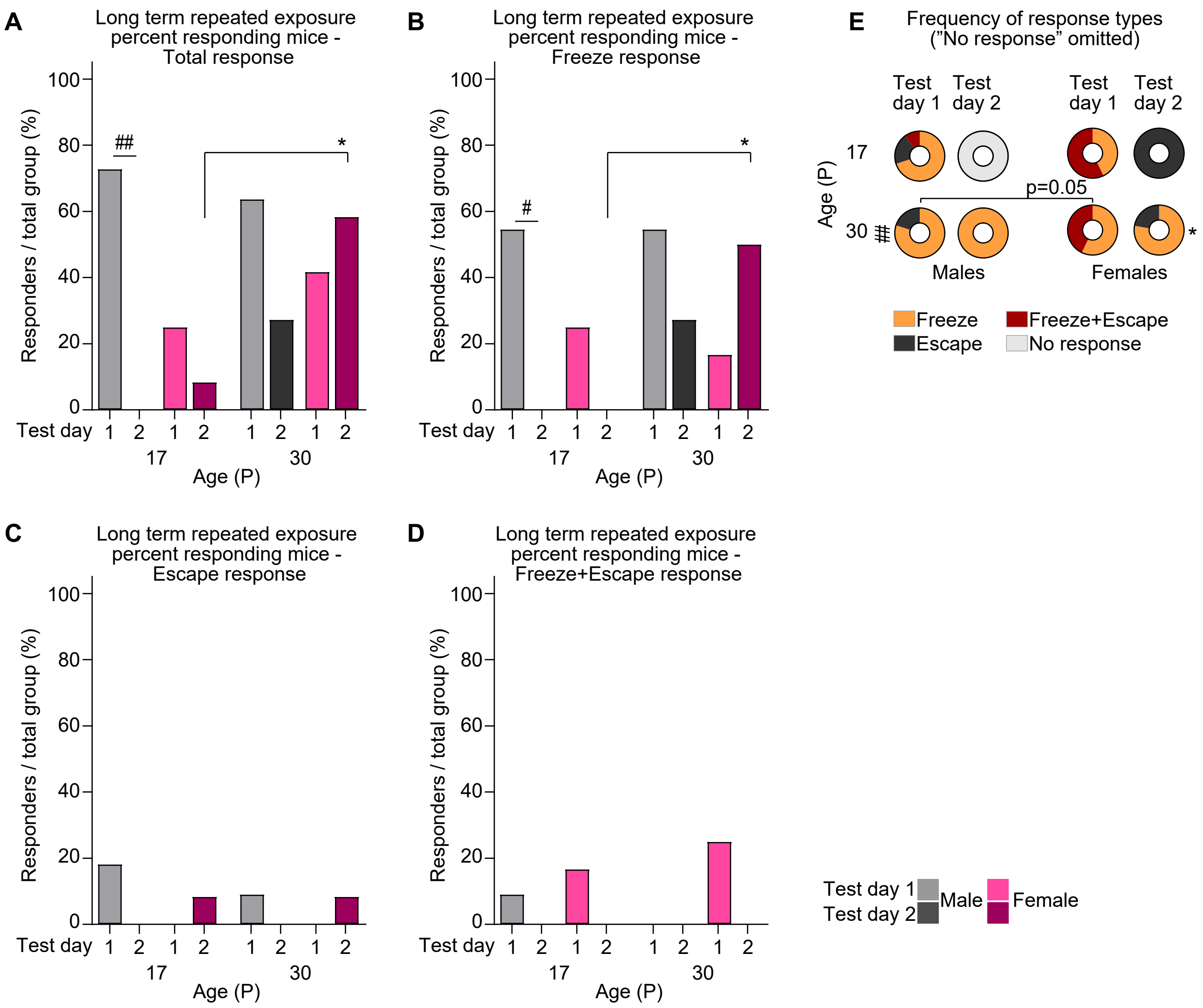
A
Long term repeated exposure percent responding mice - Total response
B
Long term repeated exposure percent responding mice - Freeze response
C
Long term repeated exposure percent responding mice - Escape response
D
Long term repeated exposure percent responding mice - Freeze+Escape response
Responders / total group (%)
100
80
60
40
20
0
##
#
*
Test day
1
2
17
30
Age (P)
E
Frequency of response types
("No response" omitted)
Test day 1
Test day 2
Age (P)
p=0.05
Males
Females
Freeze
Freeze+Escape
Escape
No response
Test day 1
Test day 2
Male
Female

Figure S3. Behavioral adaptation to the looming threat is persistent, Related to Figure 3. See also Table S1. **A-D.** Percent of responding mice to each response type as labeled. **A.** Percent responding males (Total response) is similar at P17 and P30 on Test day 1 and is reduced on Test day 2 in both age groups. Females respond at lower percentage at P17 with similar numbers at P30 on both testing days. **B.** Percent of males responding by "Freeze" is unchanged across ages and is decreased on Test day 2. Female responses increase on Test day 2 at P30. **C.** Less than 10% of male mice respond by "Escape" at P30 following initial exposure at P17. **D.** "Freeze+Escape" responding mice are almost exclusively females with ~20% responders at both ages. Graphs show percentage of mice responding to at least one trial out of total group number, see Fig. 3 for N. **E.** Doughnut plots showing the fractions of different response types from Fig. 3F, with the "No response" omitted, except in P17 males Test day 2 – where no responses were recorded (light gray). $^{*}p \leq 0.05$ comparing age groups within females; $^{\#}p \leq 0.05$, $^{\#\#}p < 0.01$, comparing age groups within males, per test day by Fisher's exact test (A-D) and Chi square Goodness-of-fit test (E). P value (*p*) on graphs indicates comparison between male and female groups by Fisher's exact test. Non-significant results ($p > 0.05$) are not shown (see Table S1).

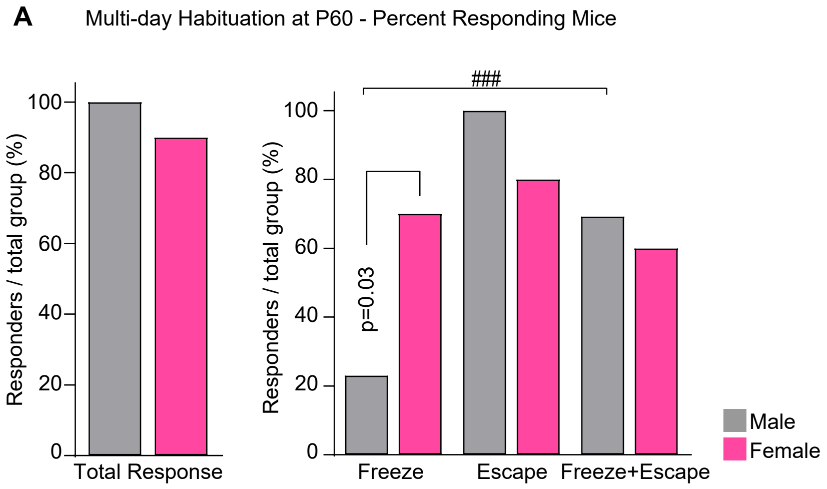
A
Multi-day Habituation at P60 - Percent Responding Mice
Responders / total group (%)
100
80
60
40
20
0
Total Response
###
p=0.03
Freeze
Escape
Freeze+Escape
Male
Female
B
Frequency of response types
(”No response” omitted)
p=0.003
###
Males
Females
Freeze
Escape
Freeze+Escape

Figure S4. Increased habituation shifts defensive response type in adult mice, Related to Figure 4. See also Table S1. **A.** Percent of responding mice out of total group number. Total responses on the left, different response types on the right. Percentage of responders by “Escape” is highest, specifically in males. Graphs show percentage, see Fig. 4 for N. **B.** Doughnut plots showing the fractions of different response types from Fig. 4C, with the “No response” omitted. $^{\#\#\#}p < 0.001$ comparing age groups within males per test day by Fisher’s exact test (A) and Chi square Goodness-of-fit test (B). P value (*p*) on graphs indicates comparison between male and female groups, by Fisher’s exact test. Non-significant results ($p > 0.05$) are not shown (see Table S1).

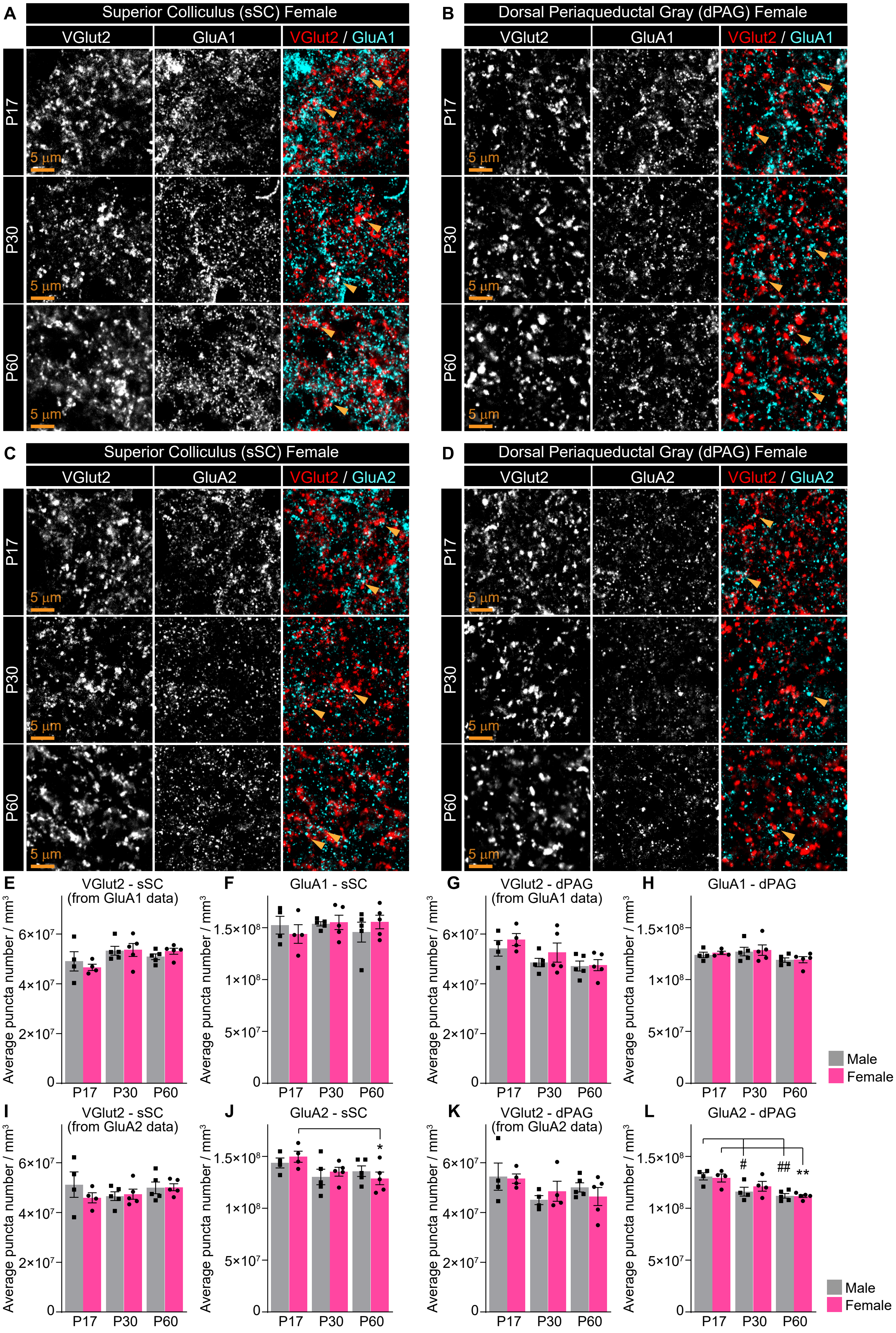
A
Superior Colliculus (sSC) Female
VGlut2
GluA1
VGlut2 / GluA1
P17
P30
P60
5 µm
B
Dorsal Periaqueductal Gray (dPAG) Female
VGlut2
GluA1
VGlut2 / GluA1
P17
P30
P60
5 µm
C
Superior Colliculus (sSC) Female
VGlut2
GluA2
VGlut2 / GluA2
P17
P30
P60
5 µm
D
Dorsal Periaqueductal Gray (dPAG) Female
VGlut2
GluA2
VGlut2 / GluA2
P17
P30
P60
5 µm
E
VGlut2 - sSC (from GluA1 data)
F
GluA1 - sSC
G
VGlut2 - dPAG (from GluA1 data)
H
GluA1 - dPAG
I
VGlut2 - sSC (from GluA2 data)
J
GluA2 - sSC
K
VGlut2 - dPAG (from GluA2 data)
L
GluA2 - dPAG
Average puncta number / mm³
0
2×10⁷
4×10⁷
6×10⁷
5×10⁷
1×10⁸
1.5×10⁸
P17
P30
P60
*
#
##
**
Male
Female

Figure S5. Developmental analysis of glutamatergic synapses in the sSC and dPAG, Related to Figure 5. See also Table S1. **A-B.** Example images of VGlut2, GluA1 and merged (synapses) in each age as labeled in the sSC (A) and dPAG (B) in female mice. **C-D.** Example images of VGlut2, GluA2 and merged (synapses) in each age as labeled in the sSC (C) and dPAG (D) in female mice. **E-H.** Quantification of VGlut2 (E, G) and GluA1 (F, H) levels represented as number of puncta per mm^3 in the sSC (E-F) and dPAG (G-H). The levels of VGlut2 and GluA1 are unaltered across the developmental time points in either sex or brain region. **I-L.** Quantification of VGlut2 (I, K) and GluA2 (J, L) levels represented as number of puncta per mm^3 in the sSC (I-J) and dPAG (K-L). The levels of VGlut2 are unaltered across the developmental time points in either sex or brain region. The levels of GluA2 are developmentally decreased in the sSC (J) and dPAG (L). Plots show mean ± s.e.m. Squares and circles indicate average of signal for each individual mouse. Number of mice/sex group (N): P17 N=4; P30, P60 N=5. Scale bar = 5 μm. Arrowheads mark representative colocalized puncta. $^{*}p \leq 0.05$, $^{**}p < 0.01$ comparing age groups within females; $^{\#}p \leq 0.05$, $^{\#\#}p < 0.01$ comparing age groups within males by one-way ANOVA. Non-significant results ($p > 0.05$) are not shown (see Table S1).

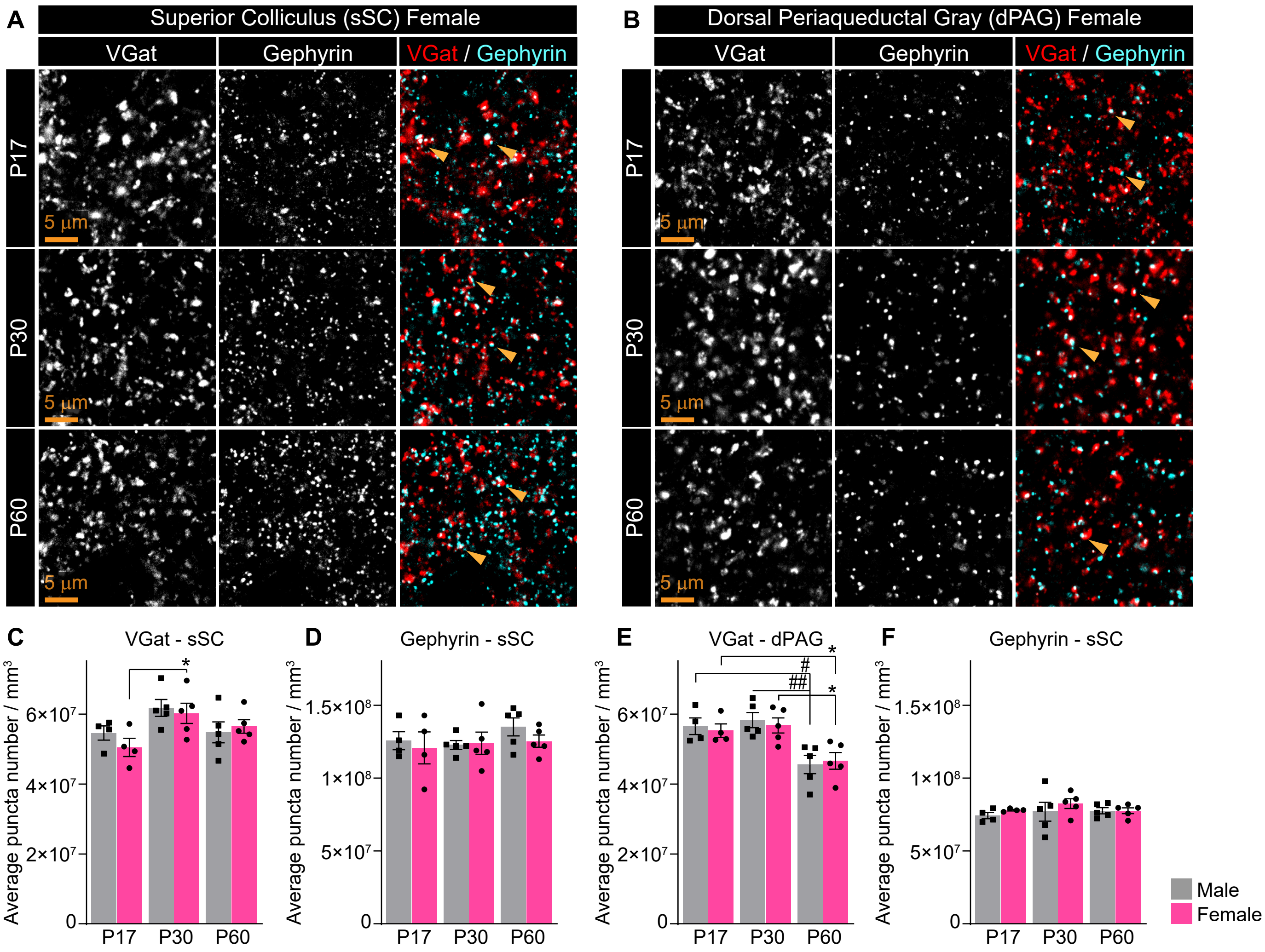
A
Superior Colliculus (sSC) Female
VGat
Gephyrin
VGat / Gephyrin
P17
P30
P60
5 µm
B
Dorsal Periaqueductal Gray (dPAG) Female
C
VGat - sSC
Average puncta number / mm3
D
Gephyrin - sSC
E
VGat - dPAG
F
Gephyrin - sSC
Male
Female

Figure S6. Developmental analysis of GABAergic synapses in the sSC and dPAG, Related to Figure 6. See also Table S1. **A-B.** Example images of VGat, Gephyrin and merged (synapses) in each age as labeled in the sSC (A) and dPAG (B) in female mice. **C-F.** Quantification of VGat (C, E) and Gephyrin (D, F) levels represented as number of puncta per mm^3 in the sSC (C-D) and dPAG (E-F). VGat levels are increased at P30 in the sSC and decreased at P60 in the dPAG compared to younger ages. The levels of Gephyrin are unchanged across all the developmental timepoints in either sex or brain region. Plots show mean ± s.e.m. Squares and circles indicate average of signal for each individual mouse. Number of mice/sex group (N): P17 N=4; P30, P60 N=5. Scale bar = 5 µm. Arrowheads mark representative colocalized puncta. $^{*}p \leq 0.05$ comparing age groups within females; $^{\#}p \leq 0.05$, $^{\#\#}p < 0.01$ comparing age groups within males by one-way ANOVA. Non-significant results ($p > 0.05$) are not shown (see Table S1).

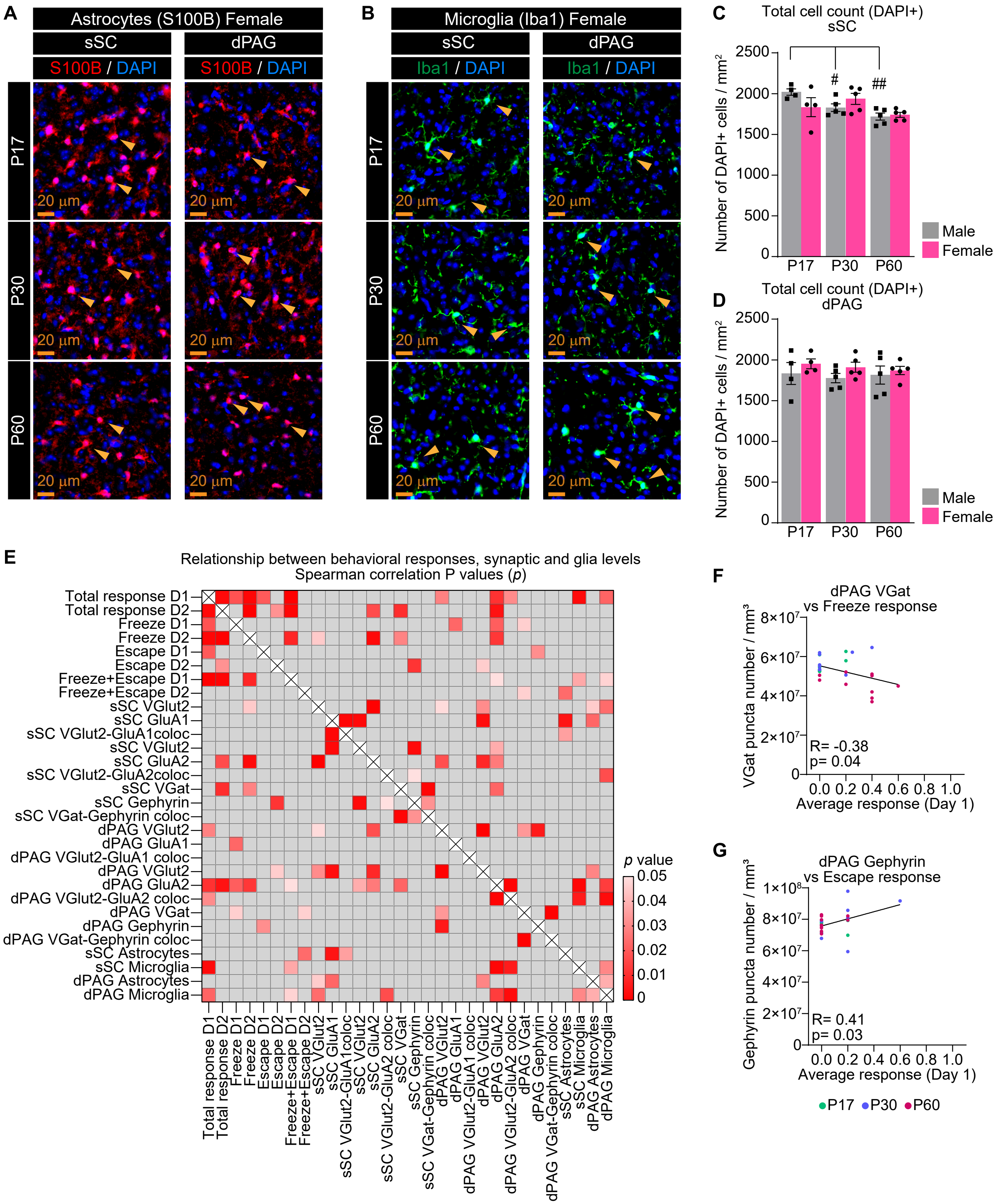
A
Astrocytes (S100B) Female
sSC
dPAG
S100B / DAPI
P17
P30
P60
20 µm
B
Microglia (Iba1) Female
sSC
dPAG
Iba1 / DAPI
P17
P30
P60
20 µm
C
Total cell count (DAPI+)
sSC
Number of DAPI+ cells / mm²
2500
2000
1500
1000
500
0
#
##
P17
P30
P60
Male
Female
D
Total cell count (DAPI+)
dPAG
Number of DAPI+ cells / mm²
2500
2000
1500
1000
500
0
P17
P30
P60
Male
Female
E
Relationship between behavioral responses, synaptic and glia levels
Spearman correlation P values (p)
Total response D1
Total response D2
Freeze D1
Freeze D2
Escape D1
Escape D2
Freeze+Escape D1
Freeze+Escape D2
sSC VGlut2
sSC GluA1
sSC VGlut2-GluA1coloc
sSC VGlut2
sSC GluA2
sSC VGlut2-GluA2coloc
sSC VGat
sSC Gephyrin
sSC VGat-Gephyrin coloc
dPAG VGlut2
dPAG GluA1
dPAG VGlut2-GluA1 coloc
dPAG VGlut2
dPAG GluA2
dPAG VGlut2-GluA2 coloc
dPAG VGat
dPAG Gephyrin
dPAG VGat-Gephyrin coloc
sSC Astrocytes
sSC Microglia
dPAG Astrocytes
dPAG Microglia
p value
0.05
0.04
0.03
0.02
0.01
0
F
dPAG VGat
vs Freeze response
VGat puncta number / mm³
8×10⁷
6×10⁷
4×10⁷
2×10⁷
0
R= -0.38
p= 0.04
0.0 0.2 0.4 0.6 0.8 1.0
Average response (Day 1)
G
dPAG Gephyrin
vs Escape response
Gephyrin puncta number / mm³
1×10⁸
8×10⁷
6×10⁷
4×10⁷
2×10⁷
0
R= 0.41
p= 0.03
0.0 0.2 0.4 0.6 0.8 1.0
Average response (Day 1)
P17
P30
P60

Figure S7

Figure S7. Developmental analysis of astrocytes and microglia in the sSC and dPAG and correlation between cellular and behavioral parameters, Related to Figure 7. See also Table S1. **A-B.** Example images of astrocytes (A) and microglia (B) in each age and brain region as labeled in female mice. **C-D.** Quantification of cell density (per mm^2) visualized by the nuclear marker DAPI in the sSC (C) and dPAG (D). Cell density is slightly decreased across development in the sSC, with no change in the dPAG for both sexes. Plots show mean ± s.e.m. Squares and circles indicate average of signal for each individual mouse. Number of mice/sex group (N): P17 N=4; P30, P60 N=5. Scale bar = 20 µm. Arrowheads mark representative S100B or Iba1 positive cells. $^{\#}p \leq 0.05$, $^{\#\#}p < 0.01$ comparing age groups within males by one-way ANOVA. Non-significant results ($p > 0.05$) are not shown. **E-G.** Spearman correlation analysis comparing histological data with behavioral responses for individual mice. **E.** Heatmap showing P values obtained by Spearman correlation analysis for the different pairwise comparisons as labeled. Significant correlations ($p \leq 0.05$) are shown in red gradient, non-significant correlations ($p > 0.05$) in gray. **F-G.** Correlation plots for pairwise comparisons as labeled showing significant negative correlation between the “Freeze” response and the inhibitory presynaptic marker VGat in the dPAG (F), and a positive correlation between the “Escape” response and the inhibitory postsynaptic marker Gephyrin (G) in the dPAG. For each comparison, Spearman R and P values (p) are shown within each plot. Datapoints are color-coded by age, P17 (green), P30 (blue), P60 (pink); N=25-27 mice.
